# Charge-Mediated Pyrin Oligomerization Nucleates Antiviral IFI16 Sensing of Herpesvirus DNA

**DOI:** 10.1128/mBio.01428-19

**Published:** 2019-07-23

**Authors:** Krystal K. Lum, Timothy R. Howard, Catherina Pan, Ileana M. Cristea

**Affiliations:** a Department of Molecular Biology, Princeton University, Princeton, New Jersey, USA; Columbia University College of Physicians & Surgeons

**Keywords:** IFI16, herpesvirus, innate immunity, mass spectrometry, oligomerization, proteomics

## Abstract

The formation of multimerized protein assemblies has emerged as a core component of immune signal amplification, yet the biochemical basis of this phenomenon remains unclear for many mammalian proteins within host defense pathways. The interferon-inducible protein 16 (IFI16) is a viral DNA sensor that oligomerizes upon binding to nuclear viral DNA and induces downstream antiviral responses. Here, we identify the pyrin domain (PYD) residues that mediate IFI16 oligomerization in a charge-dependent manner. Based on structure modeling, these residues are predicted to be surface exposed within distinct α-helices. By generating oligomerization-deficient mutants, we demonstrate that IFI16 homotypic clustering is necessary for its assembly onto parental viral genomes at the nuclear periphery upon herpes simplex virus 1 (HSV-1) infection. Preventing oligomerization severely hampered the capacity of IFI16 to induce antiviral cytokine expression, suppress viral protein levels, and restrict viral progeny production. Restoring oligomerization via residue-specific charge mimics partially rescued IFI16 antiviral roles. We show that pyrin domains from PYHIN proteins are functionally interchangeable, facilitating cooperative assembly with the IFI16 HINs, highlighting an inherent role for pyrin domains in antiviral response. Using immunoaffinity purification and targeted mass spectrometry, we establish that oligomerization promotes IFI16 interactions with proteins involved in transcriptional regulation, including PAF1C, UBTF, and ND10 bodies. We further discover PAF1C as an HSV-1 restriction factor. Altogether, our study uncovers intrinsic properties that govern IFI16 oligomerization, which serves as a signal amplification platform to activate innate immune responses and to recruit transcriptional regulatory proteins that suppress HSV-1 replication.

## Abstract (precis)

The ability of mammalian cells to detect the genomes of nuclear-replicating viruses via cellular DNA sensors is fundamental to innate immunity. Recently, mounting evidence is supporting the universal role of polymerization in these host defense factors as a signal amplification strategy. Yet, what has remained unclear are the intrinsic properties that govern their immune signal transmission. Here, we uncover the biochemical basis for oligomerization of the nuclear DNA sensor, IFI16. Upon infection with herpes simplex virus 1 (HSV-1) in human fibroblasts, we characterize the contribution of IFI16 oligomerization to downstream protein interactions and antiviral functions, including cytokine induction and suppression of HSV-1 replication. Until now, the global characterization of oligomerization-dependent protein interactions for an immune receptor has never been explored. Our integrative quantitative proteomics, molecular CRISPR/Cas9-based assays, mutational analyses, and confocal microscopy shed light on the dynamics of immune signaling cascades activated against pathogens.

## INTRODUCTION

The mammalian immune system consists of intrinsic and innate components, which act immediately to hinder pathogen invasion by recognizing pathogen- and danger-associated molecular patterns. Among this class of host defense factors is a series of evolutionarily conserved sensors (1) that can detect non-self DNA, such as viral genomes during an infection. Upon direct binding to viral genomes, DNA sensors activate both shared and distinct downstream immune and cell-homeostatic signaling cascades, which culminate in the production of proinflammatory cytokines, suppression of virus replication and spread, and regulation of cell death pathways. Within the past decade, several groups, including ours, have collectively characterized sensors that bind DNA from nuclear-replicating viruses (reviewed in references 2 and 3). Of these, the interferon-inducible protein 16 (IFI16) has emerged as a prominent DNA sensor of herpesviruses, capable of recognizing viral DNA in the nuclei of infected cells (4–6). Investigations have demonstrated that nuclear IFI16 has the capacity to contribute to inducing antiviral cytokines, including type I interferons (IFN), and suppressing viral gene expression (6–8).

Although IFI16 was shown to bind DNA of the herpesviruses herpes simplex virus 1 (HSV-1), human cytomegalovirus (HCMV), Kaposi’s sarcoma-associated herpesvirus (KSHV), and Epstein-Barr virus (EBV), its signaling mechanisms to downstream cytokine-inducing and gene silencing pathways are not fully characterized (5–10). Upon binding viral DNA via its two C-terminal HIN200 domains (HIN), IFI16 promotes cytokine expression by signaling to a central cytoplasmic axis in which the endoplasmic reticulum (ER)-resident protein stimulator of interferon genes (STING) engages the serine/threonine TANK-binding kinase 1 (TBK-1). Phosphorylated TBK-1 subsequently phosphorylates the interferon regulatory factor 3 (IRF3), inducing its dimerization and nuclear translocation (11). Nuclear IRF3 binds to IFN regulatory response elements to induce IFN-stimulated gene and cytokine expression. During HSV-1, KSHV, and EBV infections, IFI16 was also shown to form supramolecular protein complexes, inflammasomes, which facilitate the maturation of the proinflammatory cytokines, interleukin 1β (IL-1β) and IL-18 (5, 10). Additionally, upon HSV-1 infection, IFI16 suppression of viral gene expression was demonstrated to rely in part on facilitating heterochromatinization of the viral genome (8, 13). However, the mechanisms through which IFI16 initiates either immune signaling or repression of viral gene expression remain to be fully understood.

An emerging unifying characteristic of IFI16-mediated immune pathways and other DNA sensor signaling mechanisms is the formation of multimerized protein assemblies, which serve as nucleation platforms to rapidly amplify immune signals. For example, upon activation by DNA, IFI16 aggregation and filamentation have been well documented (5, 9, 13–19). This phenomenon relies upon a six α-helical pyrin domain (PYD) at the N terminus of IFI16, which is canonically known to promote homotypic protein interactions (2). Indeed, other PYD-containing proteins have been similarly observed to self-cluster, including other members of the PYD- and HIN-containing PYHIN protein family (i.e., AIM2, MNDA, and IFIX), and the inflammasome adaptor protein ASC (9, 20–22).

During nuclear DNA sensing in response to HSV-1 and HCMV infections, IFI16 oligomerization was shown to undergo distinct temporal phases, which likely represent different steps of the antiviral response. During early stages of HSV-1 and HCMV infections, the Everett group and our group observed rapid IFI16 puncta formation at sites of nuclear viral genome deposition (12, 15), and we further demonstrated that the IFI16 PYD was sufficient for this dynamic relocalization (12). A second stage of IFI16 oligomerization involves its localization to nucleoplasmic puncta containing ND10 nuclear bodies and viral replication compartments (12, 15). The relevance of IFI16 oligomerization in antiviral response was also emphasized by the identification of virus immune evasion mechanisms. During HCMV infection, the viral tegument protein pUL83 binds IFI16 (23) and inhibits PYD oligomerization (9). The IFI16 PYD is also targeted by HSV-1, as the viral E3 ubiquitin ligase ICP0 was found to promote IFI16 degradation (7, 12, 17) by specifically targeting its PYD (12). Discovering this HSV-1 immune evasion mechanism enabled investigations into IFI16 functions during active immune signaling via infection with mutant virus strains lacking ICP0 E3 ubiquitin ligase activity. These studies revealed that, when IFI16 is not targeted for degradation, its oligomerization is further expanded into fiber-like filaments later in infection (13, 17). Specifically, IFI16 filamentation was found in subsets of HSV-1 replication compartments and shown to function in attenuating RNA polymerase II association with progeny viral genomes, thereby restricting virus replication (13). Despite this knowledge, how IFI16 dynamically mediates its assembly into functional units, consisting of both early puncta and later-stage filaments, is not fully understood.

Here, we explore the intrinsic biochemical properties that promote IFI16 oligomerization and their contribution to downstream antiviral functions. Using mutational assays and microscopy, we uncoupled IFI16 oligomerization from its DNA binding ability. We demonstrate that PYD-mediated homotypic clustering is necessary for effective IFI16 assembly onto parental HSV-1 genomes at the nuclear periphery, suppression of viral protein expression, and contribution to cytokine induction. We further find that oligomerization dictates IFI16 interactions with proteins known for transcription and chromatin modulation, including the upstream binding transcription factor (UBTF) and the RNA polymerase II-associated factor 1 (PAF1) complex. Both UBTF and PAF1 colocalize with oligomeric IFI16 at HSV-1 genomes, and we uncover that PAF1 restricts HSV-1 production. Altogether, our findings provide insights into the molecular framework underlying the contributions of PYDs to protein cooperative assembly and their roles as signal amplification platforms in mammalian immune pathways.

## RESULTS

### Intermolecular charge-charge associations drive IFI16 pyrin oligomerization.

**FIG 1:**
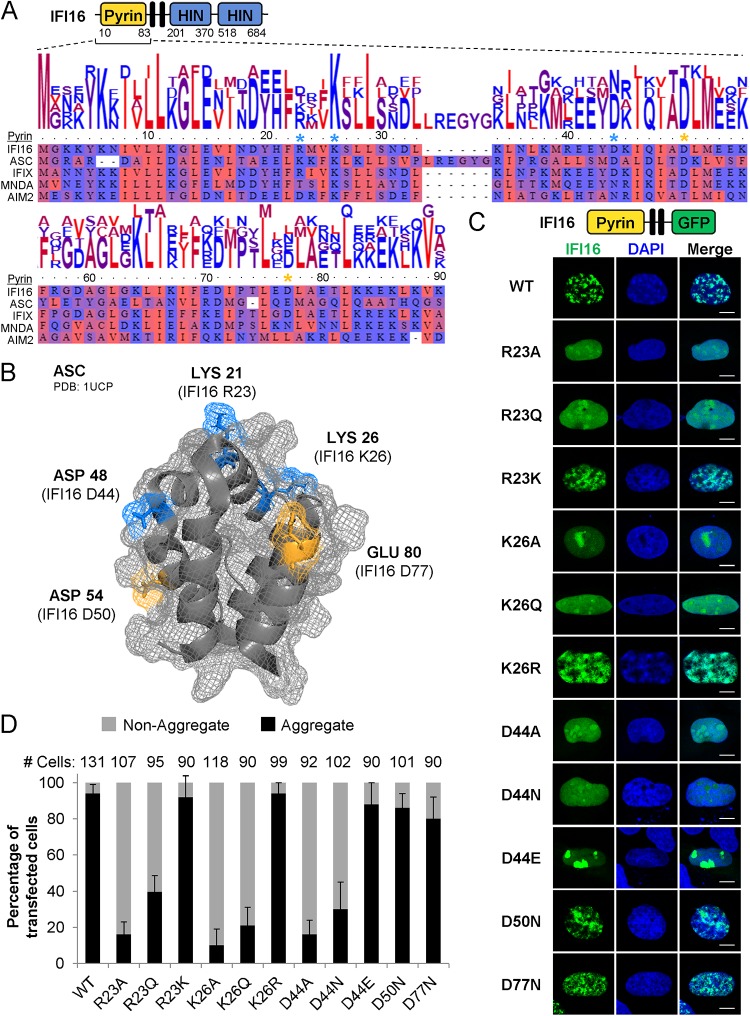
Charged residues mediate homotypic IFI16 pyrin domain aggregation. (A) Schematic of IFI16 and alignment of the PYDs of IFI16, ASC, IFIX, AIM2, and MNDA. Black bars represent the multipartite NLS. Alignment is colored by hydrophobicity. Residues predicted to be surface exposed in IFI16 (blue asterisks) and residues predicted to either impact or not impact IFI16 oligomerization (orange asterisks) are indicated. (B) NMR structure of ASC (PDB accession no. 1UCP). Charged residues shown in blue and orange sticks were selected for follow-up experimentation in IFI16. (C) Schematic of IFI16 PYD fusions to GFP. Black bars represent the NLS. Fluorescence microscopy of IFI16 PYD-GFP, bearing the indicated point mutations at R23, K26, D44, D50, and D77, in U2OS cells transfected for 24 h. Bars, 10 μm. (D) As in panel C, aggregation status was quantified in cells based on diffuse or aggregative localization of PYD-GFP. See also Fig. S1 in the supplemental material.

To identify IFI16 PYD structural properties and residues that contribute to its oligomerization, we compared the sequence homology of the IFI16 PYD with that of the pyrin-containing protein ASC and the three other mammalian PYHIN proteins, IFIX, AIM2, and MNDA (Fig. 1A). In a previous mutational screen, a series of ASC PYD residues were observed to contribute to the formation of ASC filaments (24). We observed conservation of several charged and hydrophobic regions across the PYDs of IFI16 and ASC. A majority of the hydrophobic ASC residues were buried in the three-dimensional core of the PYD, likely being involved in the proper structural folding of this domain. Therefore, we focused on charged residues R23, K26, D44, D50, and D77 that we found analogous in IFI16 (see Fig. S1A in the supplemental material).

10.1128/mBio.01428-19.2 FIG S1 Structures of ASC and MNDA PYDs. (A) NMR structure of the ASC PYD (PDB 1UCP) with hydrophobic residues known to impact filamentation (red sticks) (24). (B) Crystal structure of the MNDA PYD (PDB 5H7Q) with analogous IFI16 charged residues predicted to be surface exposed (blue and orange sticks). Download FIG S1, TIF file, 2.3 MB. Copyright © 2019 Lum et al. 2019 Lum et al. This content is distributed under the terms of the Creative Commons Attribution 4.0 International license.

As the three-dimensional structure of the IFI16 PYD is unknown, we mapped these analogous charged IFI16 residues onto the known nuclear magnetic resonance (NMR) structure of the ASC PYD (25) and observed that they reside in distinct α-helices and are all solvent exposed (Fig. 1B). In considering the observed strong electrostatic dipole moment within the ASC PYD (25), from this mapping, we hypothesized that charged, solvent-exposed residues within the IFI16 PYD contribute to its oligomerization potential. Consistent with this, upon mapping the analogous five charged residues onto the crystal structure of MNDA, we observed similar solvent exposure for each residue (Fig. S1B). Therefore, we constructed plasmids harboring wild-type (WT) PYD or a series of PYD mutants at the corresponding sites on IFI16 (R23, K26, D44, D50, and D77). All constructs contained the PYD (WT or mutant), followed by the region containing the nuclear localization signal (NLS), and were C-terminally tagged with monomeric green fluorescent protein (GFP) (PYD-GFP). As expected, expression of WT PYD-GFP generated aggregates in the nuclei of U2OS cells (Fig. 1C and D), suggesting the capacity to self-cluster. Mutations of R23, K26, and D44 to alanine perturbed this aggregation, resulting in a diffuse nuclear IFI16. Nucleolar enrichment was maintained in these alanine mutants, consistent with the localization of inactive, endogenous full-length IFI16 in cell types with predominant nuclear IFI16 (6, 7, 23, 26). Mutations of R23, K26, and D44 to structural mimics (i.e., arginine or lysine to glutamine, and aspartate to asparagine) similarly resulted in diffuse nuclear IFI16. However, charge mimic mutations at these sites (i.e., R23K, K26R, and D44E) retained the ability of IFI16 to form nuclear aggregates. For a control, to further ensure that not all structural mimic mutations of solvent-exposed sites inhibit aggregation, we also tested D50 and D77. D50N and D77N mutants formed nuclear aggregates, similar to WT IFI16 PYD, indicating that these sites may instead contribute to secondary and tertiary domain folding. These results suggest that IFI16 clustering is mediated by specific charged PYD residues, which we predict are solvent exposed in inactive IFI16 monomers. When considering previous reports on ASC, these findings further implicate that intermolecular charge-charge associations are a fundamental organizing principle for pyrin-containing proteins.

### Pyrin oligomerization mediates IFI16 association with parental HSV-1 genomes and suppression of viral replication.

Our finding of residues that support PYD aggregation provided the means to further interrogate the contribution of IFI16 self-clustering to its antiviral roles. As mentioned above, upon infection with herpesviruses, the IFI16 association with viral DNA is dynamic and temporally regulated (6, 7, 9, 12, 15, 27, 28). An initial, rapid association occurs at the nuclear periphery, where IFI16 binds to parental virus genomes being deposited into the nucleus. As we previously demonstrated that the PYD is sufficient for IFI16 relocalization to this punctate localization (12), we now asked whether PYD aggregation ability is necessary for this enrichment to incoming nuclear viral DNA during infection. We constructed full-length IFI16-GFP plasmids containing each PYD mutation at R23, K26, and D44, and inducibly expressed them in FlpIn 293s. These cells lack detectable levels of endogenous IFI16, thereby providing the ability to specifically monitor the impact of the generated mutants. Additionally, inducible expression avoids long-term exogenous IFI16 expression that can result in aggregation even in the absence of DNA substrates.

**FIG 2:**
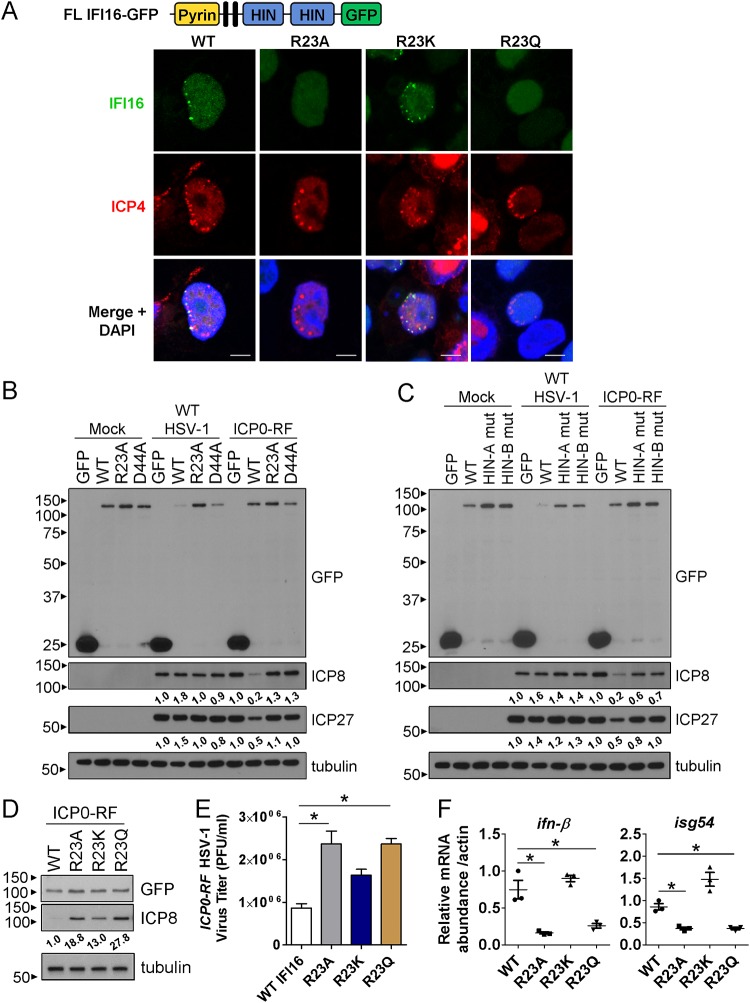
Oligomerization is required for IFI16 association with HSV-1 genomes and downstream antiviral functions. (A) Immunofluorescence microscopy of full-length IFI16-GFP, bearing mutations at R23, and ICP4. HEK293 FlpIn cells inducibly expressing the indicated IFI16-GFP fusions were infected with *ICP0-RF* HSV-1 for 24 h (multiplicity of infection [MOI] of 0.5). Representative cells are shown at the edge of a developing plaque. Bars, 5 μm. (B) Western blots of HEK293 FlpIn cells expressing the indicated IFI16-GFP fusions that affect its oligomerization state were infected with HSV-1 for 12 h (MOI of 1). Densitometry analyses of ICP8 and ICP27 are indicated relative to the GFP control and normalized to tubulin levels. (C) As in panel B with cells expressing IFI16-GFP bearing HIN mutations. (D) As in panel B with cells expressing IFI16-GFP bearing mutations at R23. (E) Progeny *ICP0-RF* HSV-1 titers (MOI of 0.2) from infected HEK293 FlpIn cells expressing IFI16-GFP R23 mutants (*n* = 3). (F) Cytokine mRNA levels in HEK293T cells cotransfected with STING and IFI16-GFP R23 mutants for 24 h. Abundances were normalized to β-actin. Values are means ± standard errors of the means (SEM) (error bars) (*n* = 3). Values that are significantly different (*P* ≤ 0.05) from those of the control by Student’s *t* test are indicated by a bar and asterisk. See also Fig. S2.

We first monitored IFI16 association with HSV-1 genomes in newly infected cells at the leading edges of a developing plaque. As WT HSV-1 targets IFI16 for degradation (7, 12, 15, 17), we infected cells with a recombinant HSV-1 strain incapable of inducing effective IFI16 degradation, *ICP0-RF*. *ICP0-RF* has mutations in the ring finger domain of the immediate early viral protein ICP0, thereby lacking its E3 ubiquitin ligase function (29). As expected, WT IFI16 was enriched in puncta asymmetrically at the inner nuclear periphery and colocalized with ICP4, a marker for early viral replication compartments (Fig. 2A; larger field developing plaque images shown in Fig. S2A). Alanine and structural mimic mutants were incapable of forming puncta, whereas charge mimics recapitulated WT IFI16 localization (Fig. 2A and Fig. S2B).

10.1128/mBio.01428-19.3 FIG S2 Analyses of IFI16 oligomerization-dependent localization and antiviral functions during HSV-1 infection. (A) Immunofluorescence microscopy of GFP and ICP4 in HEK293 FlpIn cells inducibly expressing IFI16-GFP (WT, R23A, R23K, and R23Q). Cells were infected with *ICP0-RF* HSV-1 for 24 h (MOI of 0.5). Yellow boxes indicate the representative cell(s) displayed in Fig. 2A in the main article. Bars, 100 μm. (B) Immunofluorescence microscopy as in panel A in HEK293 FlpIn cells inducibly expressing either free GFP or IFI16-GFP (WT, K26A, and D44A). Bars, 5 μm. (C) Western blot of GFP, ICP8, and ICP27 in HEK293 FlpIns inducibly expressing the indicated IFI16-GFP constructs. Cells were infected with *ICP0-RF* HSV-1 (12 hpi, MOI of 1). (D) Schematic of the HIN-A and HIN-B DNA binding mutants. (E) Western blot of GFP, STING, and tubulin in HEK293Ts transiently expressing the indicated full-length IFI16-GFP constructs and STING. (F) Western blot of GFP and tubulin in HEK293Ts transfected with the indicated IFI16-GFP constructs. Separate cell samples were both cross-linked and kept non-cross-linked for analysis. The blue arrow indicates monomeric IFI16-GFP. tub, tubulin. (G) Immunofluorescence microscopy in HEK293T cells cotransfected with IFI16-GFP (HIN domains only, WT full-length, R23A, R23K, and R23Q) and STING for 24 h. Bars, 5 μm. Perinuclear-enriched STING in cotransfected cells, as represented for WT and R23K IFI16-GFP, was quantified. Download FIG S2, TIF file, 0.7 MB. Copyright © 2019 Lum et al. 2019 Lum et al. This content is distributed under the terms of the Creative Commons Attribution 4.0 International license.

Downstream of viral genome binding, IFI16 inhibits viral protein production, a function that requires both its PYD and HIN domains (12). Therefore, we next assessed the capacity of the aggregation mutants to suppress viral protein levels. Supporting their diminished association with incoming viral DNA, alanine mutants failed to attenuate expression of the HSV-1 immediate early protein ICP27 and early protein ICP8 upon *ICP0-RF* infection (Fig. 2B and Fig. S2C). We then compared the relative contributions of IFI16 aggregation and DNA binding to its restriction of HSV-1. To accomplish this, we generated mutations in each HIN domain (HIN-A and HIN-B) of the full-length IFI16, which are mutations known to hamper IFI16 DNA binding (30, 31). The HIN-A mutant contained three sequential mutations to alanine (R370A, K371A, and K372A), whereas the HIN-B mutant contained four mutations across the domain (K571A, K676A, K678A, and K703A) (Fig. S2D). When comparing these DNA binding-deficient mutants to the aggregation-deficient mutants during infection with *ICP0-RF* virus, the loss of aggregation resulted in a more pronounced rescue of ICP8 protein levels than either of the DNA binding mutants (6.5-fold versus 3.3-fold relative to WT IFI16, respectively), suggesting a more severe loss in IFI16 antiviral activity (Fig. 2B and C).

To further assess the downstream consequences of IFI16 aggregation, we next focused on the R23 series of mutants because the charge mimic (R23K) most closely phenotypically resembled WT PYD aggregation (Fig. 1C). Additionally, we observed a significantly lower basal expression level for the K26 alanine mutant (Fig. S2C), suggesting that this highly conserved residue may be primarily structurally important, as opposed to playing a dynamic IFI16 regulatory function. Upon infection with *ICP0-RF*, expression of the R23K mutant partially rescued both the suppression of viral protein levels (Fig. 2D) and virus production (Fig. 2E), relative to R23A and R23Q IFI16. Next, we assessed the impact of these oligomerization-deficient and -capable mutants on the ability of IFI16 to induce cytokine expression. We chose to reconstitute a cytokine signaling axis by coexpressing the IFI16 constructs with STING in HEK293T cells (HEK293Ts). We chose these cells because they contain low to undetectable levels of endogenous IFI16 and STING. We confirmed equivalent levels of transfected IFI16 constructs, as well as of STING levels in these cells (Fig. S2E). To confirm IFI16 self-clustering in this context, we conducted a biochemical assay by cross-linking cell lysates and assessing the presence of high-molecular-weight IFI16 oligomers versus monomeric IFI16-GFP (Fig. S2F). As expected, we observed smears of WT and R23K IFI16-GFP at high molecular weights and did not observe any monomeric IFI16-GFP signal, which is shown in the non-cross-linked lysates. In R23A and R23Q lysates, while we observed some high-molecular-weight smears of IFI16-GFP, we also saw a strong band of monomeric IFI16-GFP, indicating ineffective self-clustering in a portion of these cells. Upon assessment of cytokines, the R23K mutant rescued the induction of *ifn-β* and *isg54* expression compared to the A and Q mutants (Fig. 2F). Consistent with these findings, we observed that only the cells expressing WT and R23K IFI16 exhibited IFI16 aggregation and an increased subset of perinuclear-enriched STING, which may correspond to enhanced STING dimerization (Fig. S2G). Altogether, these observations substantiate that nuclear IFI16 enrichment at incoming viral DNA requires PYD oligomerization and suggest that self-clustering serves as a nucleating activation signal for downstream antiviral immune responses.

### Pyrin domains from PYHIN proteins are functionally interchangeable, suggesting an inherent role for pyrin domains in antiviral response.

**FIG 3:**
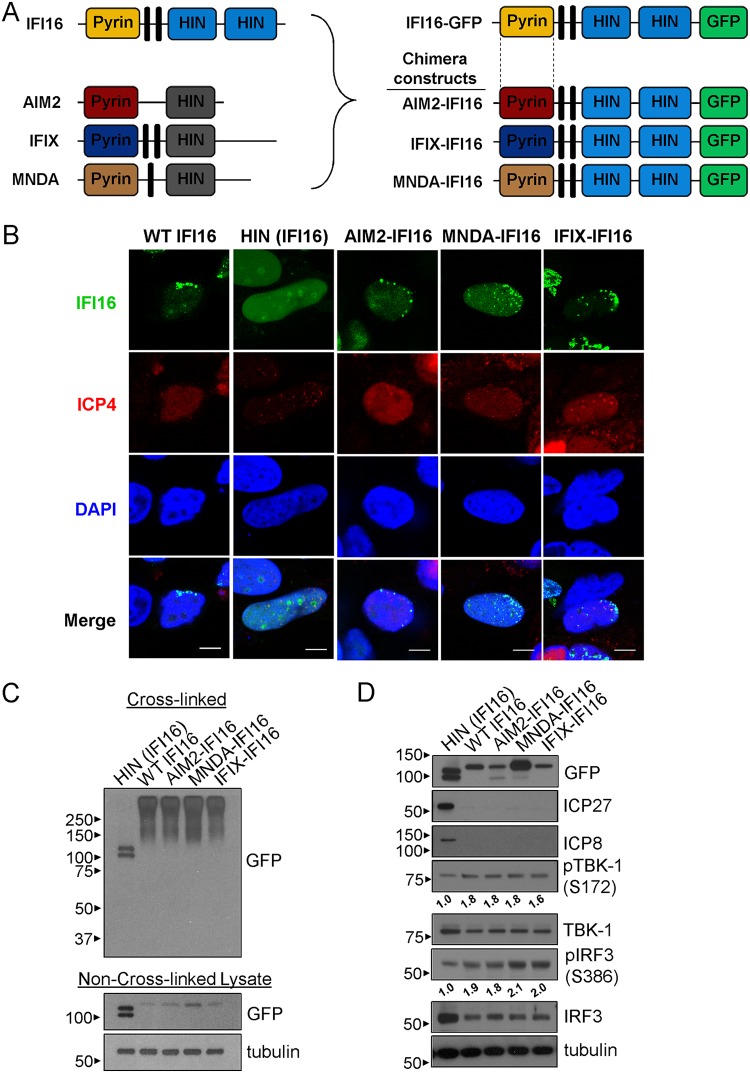
Alternative PYDs can functionally replace that of IFI16 to engage IFI16-mediated antiviral activity during HSV-1 infection. (A) Schematics of the PYHIN proteins IFI16, AIM2, IFIX, and MNDA and the generated chimera fusions to GFP. The PYD of IFI16 was swapped with that of the other PYHIN proteins. (B) Immunofluorescence microscopy of GFP and ICP4 in HEK293T cells transfected with the indicated chimera-GFP constructs. Cells were transfected and subsequently infected with *ICP0-RF* HSV-1 for 24 h (MOI of 0.5). Representative cells are shown at the edge of a developing plaque. Bars, 5 μm. (C) Western blots of HEK293Ts transfected with the indicated chimera-GFP constructs. Separate cell samples were both cross-linked and kept non-cross-linked for analysis. (D) Western blots of cells as in panel B infected with *ICP0-RF* HSV-1 for 12 h (MOI of 1). Densitometry analyses of phospho-TBK-1 and phospho-IRF3 are indicated relative to the HIN-GFP control and normalized to tubulin levels.

Pyrin-containing proteins in mammalian cells possess various degrees of phenotypic oligomerization (9, 14, 32). For example, we previously reported significant propensities for the PYDs of IFI16 and IFIX to form aggregative clusters, for that of AIM2 to form fibrous filaments, and for that of MNDA to poorly oligomerize (9). Therefore, we asked whether the IFI16 PYD can be functionally replaced by other PYDs with distinct aggregation capacities. We generated chimeric PYHIN proteins tagged with GFP by fusing the IFI16 DNA binding domains (HIN) to the PYD of AIM2, MNDA, and IFIX (Fig. 3A). Via expression in U2OS cells infected with *ICP0-RF*, cells at the edge of a developing plaque (i.e., newly infected) displayed asymmetric WT IFI16-GFP puncta at the nuclear periphery (Fig. 3B). All chimera constructs were similarly capable of forming polymerized puncta, whereas expression of the IFI16 DNA binding domains alone, HIN-GFP, could not. In agreement with this observation, cross-linking of cell lysates resulted in the appearance of high-molecular-weight oligomers and the disappearance of monomeric IFI16-GFP for cells expressing either WT IFI16-GFP or each chimera (Fig. 3C). We further showed that WT IFI16 and each chimera construct could suppress viral protein levels during *ICP0-RF* infection (Fig. 3D). For WT and each chimera, we additionally observed increased phosphorylation levels for IRF3 (at S386) and TBK-1 (at S172), markers of active immune signaling, relative to those in cells expressing the HIN domains alone. These findings are in agreement with the report of a yeast prion domain successfully replacing the PYD of the RNA sensor MAVS, as well as ASC (32), and further point to a previously unrecognized ability for all PYHIN pyrin domains to contribute to the suppression of herpesvirus protein production. Taken together, these data implicate the necessity of a polymerizing domain to function cooperatively with the DNA binding domain(s) within PYHIN proteins to activate immune responses.

### IFI16 oligomerization state promotes interactions with transcriptional modulators during HSV-1 infection.

**FIG 4:**
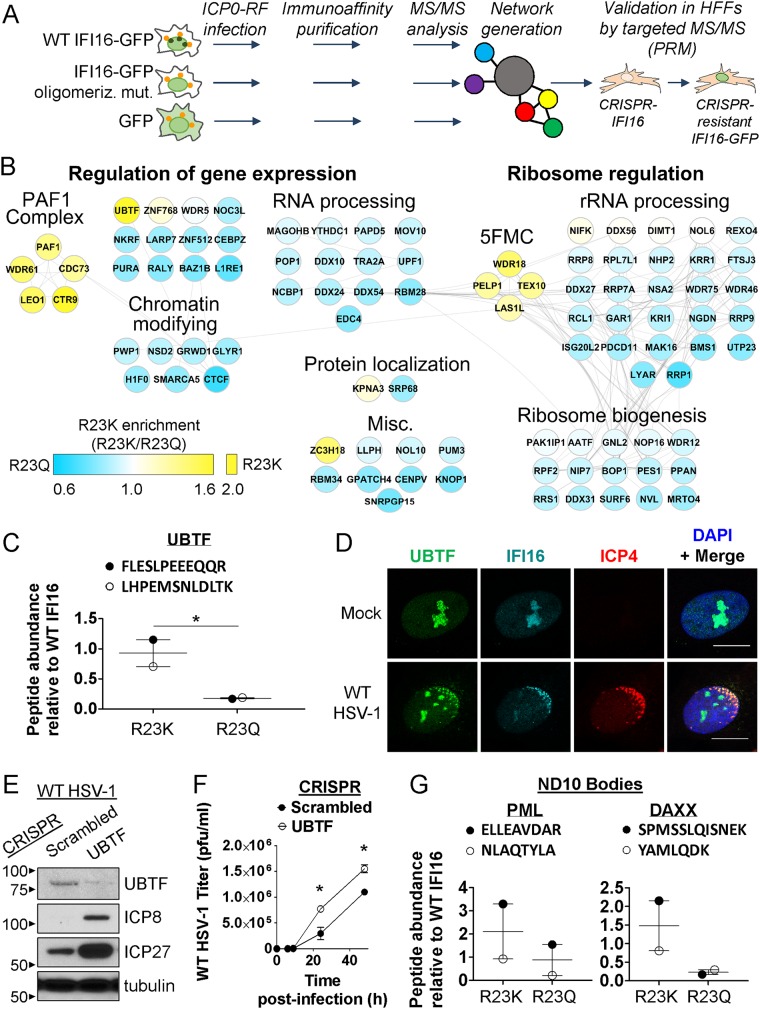
IFI16 oligomerization state dictates its interactions with transcription regulatory proteins during HSV-1 infection. (A) IP-MS/MS workflow to characterize IFI16 oligomerization-dependent protein interactions in HEK293Ts with or without *ICP0-RF* HSV-1 infection (6 hpi, MOI of 10, *n* = 2) (orange circles in cells represent viral capsids docked at the nucleus, green circles represent oligomerized IFI16 puncta at the nuclear periphery). Cells were transfected with either GFP or IFI16-GFP constructs (WT, oligomerization-competent R23K, oligomerization-deficient R23Q). Validation experiments were performed using targeted MS/MS (PRM) in CRISPR-HFFs that lacked endogenous IFI16 and stably expressed CRISPR-resistant IFI16 constructs (WT, R23K, and R23Q). In the HFF validations, the same *ICP0-RF* infection conditions were maintained. (B) Specificity-filtered IFI16 interactions were assembled into an interaction network via Cytoscape, categorized by Gene Ontology (GO) analysis. Gray lines indicate known interactions with other proteins as assessed in the Reactome database. Node colors represent relative MS^1^ protein abundances enriched with either R23K IFI16 (yellow) or R23Q IFI16 (blue). (C) Targeted PRM analysis of IFI16 oligomerization-dependent interactions with UBTF in HFFs stably expressing IFI16-GFP constructs as in panel A. Representative peptide values for each protein are relative to those of the WT IFI16 IP and normalized by average IFI16 abundance during *ICP0-RF* HSV-1 infection (6 hpi, MOI of 10). Values are means ± SEM (error bars) across peptides. Statistically significant differences were determined by one-tailed, paired Student’s *t* test (*, *P < *0.05). (D) Immunofluorescence microscopy of IFI16 and UBTF in HFFs either mock infected or infected with WT HSV-1 at 1 hpi (MOI of 10). Bars, 5 μm. (E) Western blot of ICP8 and ICP27 in CRISPR HFFs (Scrambled and UBTF) infected with WT HSV-1 at 8 hpi (MOI of 1). (F) Progeny virus titers from CRISPR HFFs (Scrambled and UBTF) infected with WT HSV-1 at 0, 6, 9, 24, and 48 hpi (MOI of 0.2; *n* = 3). Statistically significant differences were determined by unpaired Student’s *t* test (*, *P < *0.05). (G) As in panel C, upon assessment of representative peptides for ND10 body components (PML and DAXX). See also Fig. S3 and Tables S1 to S3.

A fundamental question brought forward by our findings is how oligomerization can contribute to IFI16-mediated antiviral response. Our identification of IFI16 PYD mutants that can either retain or lose association with incoming viral genomes provided us with the tools to decipher which protein interactions are specifically promoted by IFI16 oligomerization. We expressed IFI16 PYD R23Q or R23K GFP-tagged constructs in HEK293Ts. Cells were infected with *ICP0-RF* and harvested at 6 h post-infection (hpi), when expression of cytokines during HSV-1 infection was shown to be increased (7). We conducted immunoaffinity purifications (IPs) via the GFP tag and analyzed interactions (in biological duplicates) by tandem mass spectrometry (MS) (nanoscale liquid chromatography coupled to tandem mass spectrometry [nLC-MS/MS]) on a Q Exactive HF hybrid quadrupole Orbitrap mass spectrometer (Fig. 4A). Parallel control isolations in infected cells expressing GFP alone allowed the use of the computational program “significance analysis of interactome” (SAINT) (33, 50) for assessing the specificity of interactions. To further increase the stringency of the analysis, these associations were subsequently filtered by comparison with the CRAPome database (34) to remove proteins frequently observed in control isolations. The protein interactions that passed these specificity filters with either of our baits (R23Q or R23K) (see Tables S1 and S2 in the supplemental material) were placed within a functional interaction network (Fig. 4B). In agreement with our previous interaction studies of wild-type IFI16 (12, 17, 26) and known IFI16 regulatory roles, the identified associations included proteins involved in transcriptional regulation, such as in gene expression control and ribosome regulation.

10.1128/mBio.01428-19.6 TABLE S1 IFI16-GFP (R23K and R23Q) protein interactions that passed specificity filtering in HEK293Ts during *ICP0-RF* HSV-1 infection at 6 hpi (MOI of 10). Download Table S1, XLSX file, 0.04 MB. Copyright © 2019 Lum et al. 2019 Lum et al. This content is distributed under the terms of the Creative Commons Attribution 4.0 International license.

10.1128/mBio.01428-19.4 FIG S3 Immunofluorescence microscopy of PAF1, IFI16, and ICP4 in HFFs at the edge of a developing plaque during WT (MOI of 0.01) and ICP0-RF HSV-1 (MOI of 0.2) infections. Bars, 100 μm. Download FIG S3, TIF file, 2.7 MB. Copyright © 2019 Lum et al. 2019 Lum et al. This content is distributed under the terms of the Creative Commons Attribution 4.0 International license.

We next measured the relative enrichment of associations between structure and charge mimics of IFI16 PYD. To identify protein interactions enhanced by oligomerization, we focused our analysis on proteins specifically enriched with R23K relative to R23Q (Fig. 4B, yellow nodes). The interactions most highly enriched with oligomeric IFI16 were the upstream binding transcription factor (UBTF), members of two protein complexes—the RNA polymerase II-associated factor 1 (PAF1) complex and the Five Friends of Methylated Chtop (5FMC) complex, as well as the zinc finger CCCH domain-containing protein 18 (ZC3H18).

Bringing confidence to these associations, these proteins were also identified in our previous interaction studies of endogenous IFI16 or GFP-tagged WT IFI16 in fibroblasts (12, 17, 26). Therefore, we next aimed to confirm that these associations are also enriched with oligomeric IFI16 in primary human fibroblasts (HFFs). We generated CRISPR/Cas9-mediated knockouts of IFI16 in HFFs as described previously (12), as well as CRISPR-resistant forms of full-length WT IFI16-GFP or IFI16-GFP bearing the R23K and R23Q mutations. Upon stable expression of these CRISPR-resistant forms, we conducted GFP IPs in *ICP0-RF*-infected HFFs at 6 hpi. Next, we designed a quantitative targeted MS approach, based on parallel-reaction monitoring (PRM), to quantify levels of proteotypic peptides unique to each protein of interest (Fig. 4A and Table S3).

10.1128/mBio.01428-19.8 TABLE S3 Peptides targeted in GFP IPs during *ICP0-RF* HSV-1 infection (6 hpi, MOI of 10) of HFF-1s stably expressing IFI16-GFP (WT, R23K, and R23Q) or free GFP by targeted MS/MS analysis via PRM. Representative peptide values for each protein are relative to those of the WT IFI16 IP and normalized by average IFI16 abundance. Download Table S3, XLSX file, 0.01 MB. Copyright © 2019 Lum et al. 2019 Lum et al. This content is distributed under the terms of the Creative Commons Attribution 4.0 International license.

Our study of HEK293Ts pointed to UBTF as one of the two most enriched associations with oligomeric IFI16. In agreement with the HEK293T data, in HFFs, UBTF has enriched associations with R23K and WT IFI16 compared to R23Q IFI16 (Fig. 4C). This preferred association with oligomeric IFI16 may be connected to the transcriptional regulatory functions of UBTF. Like IFI16, UBTF is also known to localize to HSV-1 replication compartments and to restrict viral replication (35). By confocal microscopy, we confirmed that endogenous IFI16 and UBTF colocalize in fibroblasts during mock infection and early HSV-1 infection (Fig. 4D and Fig. S3). The infected cells displayed colocalization of UBTF and IFI16 with ICP4 at sites of HSV-1 genome entry into the nucleus. We next generated CRISPR/Cas9 knockouts of UBTF in HFFs (Fig. 4E) and observed that loss of UBTF increased viral protein levels and progeny production upon WT HSV-1 infection (Fig. 4E and F). These findings are consistent with previous observations of the antiviral impact of UBTF on HSV-1 replication in HeLa cells (35).

Among proteins implicated in transcriptional repression of virus replication, we previously characterized an association between IFI16 and components of nuclear domain 10 (ND10) bodies (12, 17, 26). ND10 body proteins, including PML and DAXX, are reported to have gene silencing roles. For example, DAXX induces a transcriptionally inactive chromatin state around the major immediate early promoter of the HCMV genome via histone deacetylase action (36). Knockdown of PML in fibroblasts was shown to increase HSV-1 and HCMV titers, recapitulating the phenotype of cells with decreased IFI16 (26, 37). Given these antiviral functions, we also probed whether IFI16 oligomerization contributes to its association with ND10 bodies. Yet, our previous IFI16 IP-MS data sets demonstrated cell type specificity for the IFI16-PML interaction, wherein we observed this association in HFFs, but not in HEK293 FlpIns (12, 17, 26). Consistent with these findings, we did not identify ND10 components in our current HEK293T interaction network (Fig. 4B). Therefore, to assess this interaction in fibroblasts, we used our CRISPR-resistant IFI16-GFP constructs in HFFs lacking endogenous IFI16, followed by IP of IFI16-GFP and PRM quantification of ND10 components. We observed an enrichment for both PML and DAXX with the oligomerization-capable R23K mutant relative to R23Q (Fig. 4G). Altogether, our work identified oligomerization-specific IFI16 interactions with several known antiviral and transcription-regulatory proteins, highlighting the importance of self-clustering for proper protein localization and function.

### PAF1 interacts with oligomerization-competent IFI16 and represses *ICP0-RF* HSV-1 progeny production.

In addition to UBTF, the other most enriched association of the IFI16 R23K mutant was with the PAF1 complex (Fig. 4B). The known core members of the complex, i.e., PAF1, CTR9, CDC73, LEO1, and WDR61 (38, 39), were found present as specific IFI16 interactions in the IP-MS data set and enriched with R23K in comparison to R23Q. Aside from its namesake in enhancing RNA polymerase II transcription elongation, PAF1C has also been shown to facilitate transcriptionally repressive H3 methylation in yeast (40). Consistent with this, PAF1C was observed to mediate H3K9 trimethylation in acute myeloid leukemia cells via interactions with the H3K9 methyltransferase, SETDB1 (41). In a small interfering RNA (siRNA) screen of cellular proteins infected with human immunodeficiency virus type 1 (HIV-1), PAF1C components (PAF1, CTR9, and RTF1), as well as SETDB1 were shown to restrict replication of the retrovirus HIV-1. PAF1C was also shown to inhibit other retroviruses, HIV-2 and simian immunodeficiency virus (SIV) infections (42).

**FIG 5:**
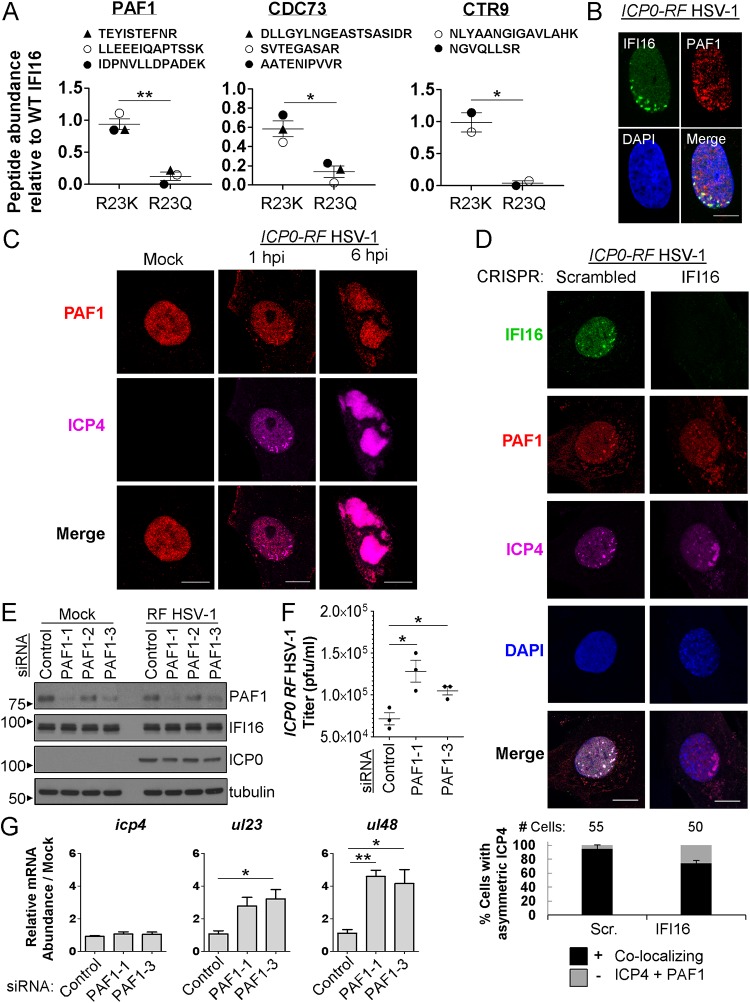
PAF1 interacts with oligomerized IFI16 and colocalizes with ICP4 to restrict HSV-1 replication. (A) Targeted PRM analysis of IFI16 oligomerization-dependent interactions with PAF1C components (PAF1, CDC73, and CTR9) in HFFs stably expressing IFI16-GFP constructs (WT, R23K, and R23Q) or free GFP. Representative peptide values for each protein are relative to those of the WT IFI16 IP and normalized by average IFI16 abundance during *ICP0-RF* HSV-1 infection (6 hpi, MOI of 10). Values are means ± SEM (error bars) across peptides. Statistically significant differences were determined by one-tailed, paired Student’s *t* test (*, *P < *0.05; **, *P < *0.005). (B) Immunofluorescence microscopy of IFI16 and PAF1 in HFFs infected with *ICP0-RF* HSV-1 at 3 hpi (MOI of 10). Bar, 10 μm. (C) Immunofluorescence microscopy of PAF1 and ICP4 in HFFs that were either mock infected or infected with *ICP0-RF* HSV-1 at 1 hpi and 6 hpi (MOI of 10). Bars, 10 μm. (D, top) Immunofluorescence microscopy of IFI16, PAF1, and ICP4 in CRISPR HFFs (Scrambled and IFI16) infected with *ICP0-RF* HSV-1 at 24 hpi (MOI of 0.2). Representative cells are shown at the edge of a developing plaque. Bars, 10 μm. (Bottom) The percentages of cells exhibiting colocalizing ICP4 and PAF1 in cells displaying nuclear, asymmetric ICP4 were quantified. (E) Western blot of PAF1 in siRNA-treated HFFs (Control and PAF1) that were either mock infected or infected with ICP0-RF HSV-1 (MOI of 10, 6 hpi). (F) Progeny virus titers in siRNA-treated HFFs as in panel E, infected with *ICP0-RF* HSV-1 (MOI of 0.2). Statistically significant differences were determined by one-tailed, unpaired Student’s *t* test (*P < *0.05). (G) Viral gene mRNA levels in siRNA-treated HFFs infected with ICP0-RF HSV-1 (MOI of 0.5) at 8 hpi. Values are means ± SEM (*n *=* *3). Statistically significant differences were determined by one-tailed, unpaired Student’s *t* test (*, *P < *0.05; **, *P < *0.01). See also Fig. S4 and Table S3.

Given the IFI16-PAF1C interaction and the known roles for IFI16 in chromatin modulation of the HSV-1 genome (8, 13), we hypothesized that PAF1C may also act as an HSV-1 restriction factor. To explore this possibility, we first confirmed the relative enrichment of PAF1, CDC73, and CTR9 with oligomerization-capable R23K, relative to oligomerization-deficient R23Q, in our CRISPR/IFI16 HFF system via IP and PRM (Fig. 5A). We were unable to detect reliable proteotypic peptides for LEO1. Yet, confirming our results from HEK293Ts, the remaining three PAF1C members reflected enriched associations with R23K relative to R23Q. The levels of the interacting partners are represented following normalization to their levels in the IP of the WT IFI16; therefore, a value of one indicates no change in interaction when comparing a mutant to WT IFI16. As expected for R23K, no significant differences were observed for PAF1 and CTR9 associations compared to WT. However, these associations were reduced for R23Q compared to either WT or R23K. CDC73 had decreased association with R23K compared to WT IFI16; yet, this association was almost lost for R23Q. Altogether, when comparing R23K and R23Q, all these decreases in associations observed for R23Q were significant. We further confirmed the colocalization of endogenous IFI16 and PAF1, the main component of the PAF1C, during *ICP0-RF* infection (Fig. 5B). From this, we observed that the site of colocalization appeared asymmetric at the nuclear periphery, suggesting possible association of both proteins with incoming HSV-1 genomes. To explore this, we assessed the colocalization of PAF1 and ICP4 at early stages of infection (1 and 6 hpi), when we and others previously observed HSV-1 genome deposition into the nucleus and the presence of viral replication compartments (12, 13, 17). We found that PAF1 colocalized with ICP4 at both infection stages, implicating PAF1 with both parental and progeny HSV-1 genomes (Fig. 5C). Next, we asked whether IFI16 promotes PAF1 association with viral genomes by monitoring PAF1 localization in CRISPR HFFs that lack IFI16 expression. We observed a decrease in PAF1 enrichment with ICP4 at the nuclear periphery in the absence of IFI16 (with approximately 25% of cells displaying decreased colocalization; Fig. 5D). In contrast, in CRISPR-control HFFs, both IFI16 and PAF1 consistently colocalized with each other and with ICP4 at the edge of a developing plaque. The absence of IFI16 did not affect PAF1 protein levels, suggesting that reduced PAF1 colocalization with ICP4 was not driven by a change in protein abundance (Fig. S4A).

10.1128/mBio.01428-19.5 FIG S4 Analyses of PAF1 levels and impact during HSV-1 infection. (A) Western blot of PAF1 levels in CRISPR-HFFs in the absence of IFI16. Cells were either mock infected or infected with *ICP0-RF* HSV-1 (MOI of 10, 1 and 6 hpi). Densitometry analyses of PAF1 are indicated relative to the CRISPR-Scrambled mock and normalized to tubulin levels. (B) Progeny virus titers in siRNA-treated HFFs, infected with WT HSV-1 (MOI of 0.2). Statistically significant differences were determined by one-tailed, unpaired Student’s *t* test (*P < *0.05). (C) Western blot of PAF1 levels in HFFs that were either mock infected or infected with WT, *ICP0-RF*, or *d106* HSV-1 (MOI of 10, 6 hpi). Download FIG S4, TIF file, 0.1 MB. Copyright © 2019 Lum et al. 2019 Lum et al. This content is distributed under the terms of the Creative Commons Attribution 4.0 International license.

Our finding that PAF1 colocalizes with viral genome compartments prompted us to assess whether PAF1 impacts HSV-1 replication. Upon siRNA-mediated knockdown of PAF1 (Fig. 5E) and HSV-1 infection, we observed increased viral progeny production (Fig. 5F and Fig. S4B). We further tested the impact of PAF1 on HSV-1 gene expression from each temporal class (*icp4*, immediate early gene; *ul23*, early gene; *ul48*, late gene; Fig. 5G). In comparison to control siRNA cells, siRNA-PAF1 cells exhibited no change in *icp4* mRNA, a partial increase in *ul23* mRNA, and a significant increase in *ul48* mRNA levels. In agreement, ICP0 protein levels were unchanged upon PAF1 knockdown (Fig. S4C). Altogether, our results uncover that IFI16 and PAF1 colocalize with HSV-1 genomes and that PAF1 may restrict HSV-1 production by affecting late viral gene expression.

### Concluding remarks.

The ability of DNA sensors to bind pathogen-derived DNA and immediately amplify immune signals is essential for efficient restriction of pathogen replication and spread. In recent years, mounting evidence is supporting the universal prevalence of polymerization in these innate host defense factors as a signal amplification platform. Yet, what has remained unclear are the intrinsic properties of most DNA sensors that govern their activation and immune signal transmission. In this study, we explored the biochemistry of nuclear IFI16 oligomerization and its impact on downstream immune response functions against nuclear-replicating HSV-1. On the basis of our findings, we propose that upon binding to HSV-1 DNA deposited into the nucleus, IFI16 PYDs facilitate dynamic, local clustering of IFI16-DNA complexes into puncta via intermolecular associations between surface-exposed charged residues. We expect that these residues mediate both IFI16 punctum formation at the nuclear periphery early in infection and the later filamentation at viral replication compartments. These oligomeric activities further promote the downstream suppression of HSV-1 protein levels and replication, as well as contributions to antiviral cytokine expression.

Our results suggest that mammalian PYDs can serve as distinct functional units and have at least one conserved capacity to engage in cooperative assembly with C-terminal DNA binding HIN domains. In support of this, Gray et al. reported that a chimeric construct containing the AIM2 PYD and IFI16 HINs induced canonical AIM2-specific pyroptotic cell death to an extent similar to that of WT AIM2 (43). Yet, chimeras of the AIM2 PYD with either the MNDA or IFIX HINs poorly induced cell death. Considering that we previously showed that IFIX binds interferon-stimulating dsDNA and HSV-1 genomes (26, 44), these results suggest that AIM2 PYD-mediated activity requires a threshold of dsDNA binding affinity that was not achieved by either the MNDA or IFIX HINs. Furthermore, our study demonstrates that the respective PYDs remain capable of conferring IFI16-specific functions when associated with active IFI16 HINs. Accounts in the literature of PYD-only proteins having distinct roles, such as inhibition of inflammasomes (45–47), further reinforce these interpretations.

From our explorations into IFI16 protein interactions, we identified several oligomerization-promoted associations, including with the 5FMC complex, ND10 body components, UBTF, and PAF1C. These IFI16 associations are possibly functionally related to the known transcriptional roles of these complexes. Knockdown of PELP1 or SENP3 was reported to decrease HSV-1 titers (44), and PELP1 was found enriched with chromatin during HSV-1 infection at 8 hpi relative to mock infection (48). These data implicate a transcription-activating capacity for the 5FMC at HSV-1 genomes in replication compartments. The IFI16 association with ND10 body components is also an area of active research. Most recently, it was reported that IFI16 recruits ND10 components (e.g., PML, Sp100, ATRX) to HSV-1 DNA for virus restriction (13). Our lab previously observed that depletion of PML isoform I via CRISPR did not impact cytokine production (12). These findings suggest that the IFI16-ND10 association is perhaps functionally important for restricting HSV-1 replication, rather than cytokine production. Yet, others report that ND10 body association with incoming HSV-1 genomes and viral restriction is independent of IFI16 in the absence of ICP0 (49). As such, future experimentation is required to explore these interactions.

Our study further uncovered PAF1 as a likely virus restriction factor during HSV-1 infection. Considering that both UBTF and PAF1C consist of proteins that increase virus titers when knocked down in cells, it is possible that these IFI16 interactions may act in concert to transcriptionally repress viral gene expression. Our additional observation that in a subset of infected cells lacking IFI16, PAF1 did not colocalize with ICP4 at the edge of developing plaques suggests several possibilities. One explanation is that PAF1 can localize to viral genomes, which is aided by an initial IFI16-DNA association but does not require or is not recruited by IFI16. Another possibility is that coordination between IFI16 and PAF1C exists to repress viral replication in an accessory fashion with one another. Alternatively, in the absence of IFI16, the kinetics of the global host antiviral response may be delayed such that lytic replication progresses faster than under control conditions. Additionally, this assay inherently generates cells at different stages of infection, which must be acknowledged with caution when interpreting results. Considering that the dynamics of PAF1 localization during HSV-1 infection have yet to be studied, future live time-lapse microscopy experiments will be of great value to determine the relationship of PAF1, IFI16, and HSV-1 restriction.

Taken together, our work provides support for the notion that oligomerization is a conserved and critical strategy used to amplify immune signaling in innate immune receptors, helping to shape the outcome of pathogen infection. We envision that understanding the basis for oligomerization in immune proteins will be critical for developing immunotherapies and antiviral therapeutics, as well as disaggregase treatments for autoimmune diseases associated with interferonopathies.

## MATERIALS AND METHODS

Complete descriptions of the materials and methods are provided in Text S1 in the supplemental material.

10.1128/mBio.01428-19.1 TEXT S1 Supplemental materials and methods. Download Text S1, DOCX file, 0.03 MB. Copyright © 2019 Lum et al. 2019 Lum et al. This content is distributed under the terms of the Creative Commons Attribution 4.0 International license.

### Cell line construction, transfections, and lentivirus.

To generate cell lines, lentiviruses (pLEX-MCS and LentiCRISPRv2) were prepared according to protocols from the RNA interference (RNAi) Consortium. For CRISPR, candidate 20-bp guide RNA sequences were designed (as in http://crispr.mit.edu/) and delivered using the LentiCRISPRv2 vector (50) (Addgene plasmid 52961) from Feng Zhang. Lentiviruses were generated in and harvested from HEK293T cells: packaging vectors psPAX2 and pMD2.G (VSV-G) were cotransfected with a lentiviral transfer vector using XtremeGENE HP transfection reagent (Roche Diagnostics).

### Fluorescence imaging.

For immunofluorescence imaging, fixed and permeabilized cells were sequentially probed with primary antibody and Alexa Fluor fluorophore-conjugated secondary antibody (Life Technologies). Microscopy was performed using a Leica TC SP5 confocal microscope (Leica Microsystems) and an inverted fluorescence confocal microscope (Nikon Ti-E) equipped with a Yokogawa spinning disc (CSU-21) and digital camera (Hamamatsu ORCA-Flash TuCam).

### IFI16-GFP IP and identification of interactions by MS.

IFI16-GFP immunoaffinity purifications (IPs) were carried out using GFP-Trap_MA GFP antibody-coupled magnetic beads (Chromotek). IP eluates were trypsinized in suspension trapping columns (S-Trap; Protifi). Resulting peptides were analyzed by nLC-MS/MS with a Q Exactive HF Hybrid Quadrupole Orbitrap instrument (Thermo Fisher Scientific) using data-dependent acquisition (DDA) or parallel-reaction monitoring (PRM) modes. MS/MS spectra were analyzed by Proteome Discoverer v2.2 (Thermo Fisher Scientific). MS spectra were searched using the Sequest HT algorithm against a UniProt human database containing herpesvirus sequences and common contaminants. Identified proteins were filtered for specificity with the Significance Analysis of INTeractome express (SAINTexpress) algorithm (33, 51) using MS^1^ intensity values with a cutoff score of 0.80 (see Table S1 in the supplemental material). Then, proteins were cross-referenced against the CRAPome repository (34) to further filter for specific interactions; proteins appearing in >20% of control experiments were removed (Table S2). Specificity-filtered proteins were used to generate an interaction network using the Cytoscape software (v. 3.7.0) (52), and proteins were categorized by Gene Ontology.

10.1128/mBio.01428-19.7 TABLE S2 List of putative GFP-IFI16 interactions in HEK293Ts excluded based on high-frequency presence in the CRAPome repository. Download Table S2, XLSX file, 0.02 MB. Copyright © 2019 Lum et al. 2019 Lum et al. This content is distributed under the terms of the Creative Commons Attribution 4.0 International license.

PRM assays were designed and analyzed using the Skyline Daily software (53). Proteotypic peptides for each protein of interest were selected, and the elution and fragmentation profiles were experimentally determined for each targeted peptide (Table S3). Peptide abundance was quantified using the summed area under the curve of three to five fragment ions per peptide.

### Data availability.

The MS proteomics data, including the MS raw files, have been deposited in the ProteomeXchange Consortium repository via the PRIDE partner repository (54). The accession number for the MS proteomic data is ProteomeXchange PXD014349. PRM data have been deposited into Panorama Public (https://panoramaweb.org/90ay43.url).

## References

[1] Gallucci S, Maffei ME 2017 DNA sensing across the tree of life. Trends Immunol 38:719–732. doi:10.1016/j.it.2017.07.012. 28886908

[2] Diner BA, Lum KK, Cristea IM 2015 The emerging role of nuclear viral DNA sensors. J Biol Chem 290:26412–26421. doi:10.1074/jbc.R115.652289. 26354430 PMC4646299

[3] Abe T, Marutani Y, Shoji I 2019 Cytosolic DNA-sensing immune response and viral infection. Microbiol Immunol 63:51. doi:10.1111/1348-0421.12669. 30677166 PMC7168513

[4] Unterholzner L, Keating SE, Baran M, Horan KA, Jensen SB, Sharma S, Sirois CM, Jin TC, Latz E, Xiao TS, Fitzgerald KA, Paludan SR, Bowie AG 2010 IFI16 is an innate immune sensor for intracellular DNA. Nat Immunol 11:997. doi:10.1038/ni.1932. 20890285 PMC3142795

[5] Kerur N, Veettil MV, Sharma-Walia N, Bottero V, Sadagopan S, Otageri P, Chandran B 2011 IFI16 acts as a nuclear pathogen sensor to induce the inflammasome in response to Kaposi sarcoma-associated herpesvirus infection. Cell Host Microbe 9:363–375. doi:10.1016/j.chom.2011.04.008. 21575908 PMC3113467

[6] Li T, Diner BA, Chen J, Cristea IM 2012 Acetylation modulates cellular distribution and DNA sensing ability of interferon-inducible protein IFI16. Proc Natl Acad Sci U S A 109:10558–10563. doi:10.1073/pnas.1203447109. 22691496 PMC3387042

[7] Orzalli MH, DeLuca NA, Knipe DM 2012 Nuclear IFI16 induction of IRF-3 signaling during herpesviral infection and degradation of IFI16 by the viral ICP0 protein. Proc Natl Acad Sci U S A 109:E3008–E3017. doi:10.1073/pnas.1211302109. 23027953 PMC3497734

[8] Orzalli MH, Conwell SE, Berrios C, DeCaprio JA, Knipe DM 2013 Nuclear interferon-inducible protein 16 promotes silencing of herpesviral and transfected DNA. Proc Natl Acad Sci U S A 110:E4492–E4501. doi:10.1073/pnas.1316194110. 24198334 PMC3839728

[9] Li T, Chen J, Cristea IM 2013 Human cytomegalovirus tegument protein pUL83 inhibits IFI16-mediated DNA sensing for immune evasion. Cell Host Microbe 14:591–599. doi:10.1016/j.chom.2013.10.007. 24237704 PMC3876934

[10] Ansari MA, Singh VV, Dutta S, Veettil MV, Dutta D, Chikoti L, Lu J, Everly D, Chandran B 2013 Constitutive interferon-inducible protein 16-inflammasome activation during Epstein-Barr virus latency I, II, and III in B and epithelial cells. J Virol 87:8606–8623. doi:10.1128/JVI.00805-13. 23720728 PMC3719826

[11] Hornung V, Latz E 2010 Intracellular DNA recognition. Nat Rev Immunol 10:123–130. doi:10.1038/nri2690. 20098460

[12] Diner BA, Lum KK, Toettcher JE, Cristea IM 2016 Viral DNA sensors IFI16 and cyclic GMP-AMP synthase possess distinct functions in regulating viral gene expression, immune defenses, and apoptotic responses during herpesvirus infection. mBio 7:e01553-16. doi:10.1128/mBio.01553-16. 27935834 PMC5111403

[13] Merkl PE, Knipe DM 2019 Role for a filamentous nuclear assembly of IFI16, DNA, and host factors in restriction of herpesviral infection. mBio 10:e02621-18. doi:10.1128/mBio.02621-18. 30670617 PMC6343039

[14] Hornung V, Ablasser A, Charrel-Dennis M, Bauernfeind F, Horvath G, Caffrey DR, Latz E, Fitzgerald KA 2009 AIM2 recognizes cytosolic dsDNA and forms a caspase-1-activating inflammasome with ASC. Nature 458:514–518. doi:10.1038/nature07725. 19158675 PMC2726264

[15] Cuchet-Lourenco D, Anderson G, Sloan E, Orr A, Everett RD 2013 The viral ubiquitin ligase ICP0 is neither sufficient nor necessary for degradation of the cellular DNA sensor IFI16 during herpes simplex virus 1 infection. J Virol 87:13422–13432. doi:10.1128/JVI.02474-13. 24089555 PMC3838218

[16] Morrone SR, Wang T, Constantoulakis LM, Hooy RM, Delannoy MJ, Sohn J 2014 Cooperative assembly of IFI16 filaments on dsDNA provides insights into host defense strategy. Proc Natl Acad Sci U S A 111:E62–E71. doi:10.1073/pnas.1313577111. 24367117 PMC3890864

[17] Diner BA, Lum KK, Javitt A, Cristea IM 2015 Interactions of the antiviral factor interferon gamma-inducible protein 16 (IFI16) mediate immune signaling and herpes simplex virus-1 immunosuppression. Mol Cell Proteomics 14:2341–2356. doi:10.1074/mcp.M114.047068. 25693804 PMC4563720

[18] Stratmann SA, Morrone SR, van Oijen AM, Sohn J 2015 The innate immune sensor IFI16 recognizes foreign DNA in the nucleus by scanning along the duplex. Elife 4:e11721. doi:10.7554/eLife.11721. 26673078 PMC4829420

[19] Antiochos B, Matyszewski M, Sohn J, Casciola-Rosen L, Rosen A 2018 IFI16 filament formation in salivary epithelial cells shapes the anti-IFI16 immune response in Sjogren’s syndrome. JCI Insight 3:120179. doi:10.1172/jci.insight.120179. 30232276 PMC6237237

[20] Fernandes-Alnemri T, Yu JW, Datta P, Wu J, Alnemri ES 2009 AIM2 activates the inflammasome and cell death in response to cytoplasmic DNA. Nature 458:509–513. doi:10.1038/nature07710. 19158676 PMC2862225

[21] Lu A, Magupalli VG, Ruan J, Yin Q, Atianand MK, Vos MR, Schroder GF, Fitzgerald KA, Wu H, Egelman EH 2014 Unified polymerization mechanism for the assembly of ASC-dependent inflammasomes. Cell 156:1193–1206. doi:10.1016/j.cell.2014.02.008. 24630722 PMC4000066

[22] Dick MS, Sborgi L, Ruhl S, Hiller S, Broz P 2016 ASC filament formation serves as a signal amplification mechanism for inflammasomes. Nat Commun 7:11929. doi:10.1038/ncomms15030. 27329339 PMC4917984

[23] Cristea IM, Moorman NJ, Terhune SS, Cuevas CD, O’Keefe ES, Rout MP, Chait BT, Shenk T 2010 Human cytomegalovirus pUL83 stimulates activity of the viral immediate-early promoter through its interaction with the cellular IFI16 protein. J Virol 84:7803–7814. doi:10.1128/JVI.00139-10. 20504932 PMC2897612

[24] Moriya M, Taniguchi S, Wu P, Liepinsh E, Otting G, Sagara J 2005 Role of charged and hydrophobic residues in the oligomerization of the PYRIN domain of ASC. Biochemistry 44:575–583. doi:10.1021/bi048374i. 15641782

[25] Liepinsh E, Barbals R, Dahl E, Sharipo A, Staub E, Otting G 2003 The death-domain fold of the ASC PYRIN domain, presenting a basis for PYRIN/PYRIN recognition. J Mol Biol 332:1155–1163. doi:10.1016/j.jmb.2003.07.007. 14499617

[26] Diner BA, Li T, Greco TM, Crow MS, Fuesler JA, Wang J, Cristea IM 2015 The functional interactome of PYHIN immune regulators reveals IFIX is a sensor of viral DNA. Mol Syst Biol 11:787. doi:10.15252/msb.20145808. 25665578 PMC4358659

[27] Johnson KE, Bottero V, Flaherty S, Dutta S, Singh VV, Chandran B 2014 IFI16 restricts HSV-1 replication by accumulating on the HSV-1 genome, repressing HSV-1 gene expression, and directly or indirectly modulating histone modifications. PLoS Pathog 10:e1004503. doi:10.1371/journal.ppat.1004503. 25375629 PMC4223080

[28] Everett RD 2016 Dynamic response of IFI16 and promyelocytic leukemia nuclear body components to herpes simplex virus 1 infection. J Virol 90:167–179. doi:10.1128/JVI.02249-15. 26468536 PMC4702556

[29] Lium EK, Silverstein S 1997 Mutational analysis of the herpes simplex virus type 1 ICP0 C3HC4 zinc ring finger reveals a requirement for ICP0 in the expression of the essential alpha27 gene. J Virol 71:8602–8614. 9343218 10.1128/jvi.71.11.8602-8614.1997 PMC192324

[30] Jin TC, Perry A, Jiang JS, Smith P, Curry JA, Unterholzner L, Jiang ZZ, Horvath G, Rathinam VA, Johnstone RW, Hornung V, Latz E, Bowie AG, Fitzgerald KA, Xiao TS 2012 Structures of the HIN domain: DNA complexes reveal ligand binding and activation mechanisms of the AIM2 inflammasome and IFI16 receptor. Immunity 36:561–571. doi:10.1016/j.immuni.2012.02.014. 22483801 PMC3334467

[31] Ni XM, Ru H, Ma F, Zhao LX, Shaw N, Feng YG, Ding W, Gong WB, Wang QF, Ouyang SY, Cheng GH, Liu ZJ 2016 New insights into the structural basis of DNA recognition by HINa and HINb domains of IFI16. J Mol Cell Biol 8:51–61. doi:10.1093/jmcb/mjv053. 26246511

[32] Cai X, Chen JQ, Xu H, Liu SQ, Jiang QX, Halfmann R, Chen Z 2014 Prion-like polymerization underlies signal transduction in antiviral immune defense and inflammasome activation. Cell 156:1207–1222. doi:10.1016/j.cell.2014.01.063. 24630723 PMC4034535

[33] Choi H, Larsen B, Lin ZY, Breitkreutz A, Mellacheruvu D, Fermin D, Qin ZS, Tyers M, Gingras AC, Nesvizhskii AI 2011 SAINT: probabilistic scoring of affinity purification-mass spectrometry data. Nat Methods 8:70–73. doi:10.1038/nmeth.1541. 21131968 PMC3064265

[34] Mellacheruvu D, Wright Z, Couzens AL, Lambert JP, St-Denis NA, Li T, Miteva YV, Hauri S, Sardiu ME, Low TY, Halim VA, Bagshaw RD, Hubner NC, Al-Hakim A, Bouchard A, Faubert D, Fermin D, Dunham WH, Goudreault M, Lin ZY, Badillo BG, Pawson T, Durocher D, Coulombe B, Aebersold R, Superti-Furga G, Colinge J, Heck AJR, Choi H, Gstaiger M, Mohammed S, Cristea IM, Bennett KL, Washburn MP, Raught B, Ewing RM, Gingras AC, Nesvizhskii AI 2013 The CRAPome: a contaminant repository for affinity purification-mass spectrometry data. Nat Methods 10:730–736. doi:10.1038/nmeth.2557. 23921808 PMC3773500

[35] Lavallee GO, Pearson A 2015 Upstream binding factor inhibits herpes simplex virus replication. Virology 483:108–116. doi:10.1016/j.virol.2015.04.003. 25965800

[36] Newhart A, Rafalska-Metcalf IU, Yang T, Negorev DG, Janicki SM 2012 Single-cell analysis of Daxx and ATRX-dependent transcriptional repression. J Cell Sci 125:5489–5501. doi:10.1242/jcs.110148. 22976303 PMC3561858

[37] Tavalai N, Papior P, Rechter S, Leis M, Stamminger T 2006 Evidence for a role of the cellular ND10 protein PML in mediating intrinsic immunity against human cytomegalovirus infections. J Virol 80:8006–8018. doi:10.1128/JVI.00743-06. 16873257 PMC1563799

[38] Jaehning JA 2010 The Paf1 complex: platform or player in RNA polymerase II transcription? Biochim Biophys Acta 1799:379–388. doi:10.1016/j.bbagrm.2010.01.001. 20060942 PMC2862274

[39] Kim J, Guermah M, Roeder RG 2010 The human PAF1 complex acts in chromatin transcription elongation both independently and cooperatively with SII/TFIIS. Cell 140:491–503. doi:10.1016/j.cell.2009.12.050. 20178742 PMC2853908

[40] Krogan NJ, Dover J, Wood A, Schneider J, Heidt J, Boateng MA, Dean K, Ryan OW, Golshani A, Johnston M, Greenblatt JF, Shilatifard A 2003 The Paf1 complex is required for histone H3 methylation by COMPASS and Dot1p: linking transcriptional elongation to histone methylation. Mol Cell 11:721–729. doi:10.1016/S1097-2765(03)00091-1. 12667454

[41] Ropa J, Saha N, Chen Z, Serio J, Chen W, Mellacheruvu D, Zhao L, Basrur V, Nesvizhskii AI, Muntean AG 2018 PAF1 complex interactions with SETDB1 mediate promoter H3K9 methylation and transcriptional repression of Hoxa9 and Meis1 in acute myeloid leukemia. Oncotarget 9:22123–22136. doi:10.18632/oncotarget.25204. 29774127 PMC5955148

[42] Liu L, Oliveira NM, Cheney KM, Pade C, Dreja H, Bergin AM, Borgdorff V, Beach DH, Bishop CL, Dittmar MT, McKnight A 2011 A whole genome screen for HIV restriction factors. Retrovirology 8:94. doi:10.1186/1742-4690-8-94. 22082156 PMC3228845

[43] Gray EE, Winship D, Snyder JM, Child SJ, Geballe AP, Stetson DB 2016 The AIM2-like receptors are dispensable for the interferon response to intracellular DNA. Immunity 45:255–266. doi:10.1016/j.immuni.2016.06.015. 27496731 PMC4988931

[44] Crow MS, Cristea IM 2017 Human antiviral protein IFIX suppresses viral gene expression during herpes simplex virus 1 (HSV-1) infection and is counteracted by virus-induced proteasomal degradation. Mol Cell Proteomics 16:S200–S214. doi:10.1074/mcp.M116.064741. 28077445 PMC5393386

[45] Ratsimandresy RA, Chu LH, Khare S, de Almeida L, Gangopadhyay A, Indramohan M, Misharin AV, Greaves DR, Perlman H, Dorfleutner A, Stehlik C 2017 The PYRIN domain-only protein POP2 inhibits inflammasome priming and activation. Nat Commun 8:15556. doi:10.1038/ncomms15556. 28580931 PMC5465353

[46] de Almeida L, Khare S, Misharin AV, Patel R, Ratsimandresy RA, Wallin MC, Perlman H, Greaves DR, Hoffman HM, Dorfleutner A, Stehlik C 2015 The PYRIN domain-only protein POP1 inhibits inflammasome assembly and ameliorates inflammatory disease. Immunity 43:264–276. doi:10.1016/j.immuni.2015.07.018. 26275995 PMC4666005

[47] Johnston JB, Barrett JW, Nazarian SH, Goodwin M, Ricciuto D, Wang G, McFadden G 2005 A poxvirus-encoded pyrin domain protein interacts with ASC-1 to inhibit host inflammatory and apoptotic responses to infection. Immunity 23:587–598. doi:10.1016/j.immuni.2005.10.003. 16356857

[48] Kulej K, Avgousti DC, Sidoli S, Herrmann C, Della Fera AN, Kim ET, Garcia BA, Weitzman MD 2017 Time-resolved global and chromatin proteomics during herpes simplex virus type 1 (HSV-1) infection. Mol Cell Proteomics 16:S92–S107. doi:10.1074/mcp.M116.065987. 28179408 PMC5393384

[49] Alandijany T, Roberts APE, Conn KL, Loney C, McFarlane S, Orr A, Boutell C 2018 Distinct temporal roles for the promyelocytic leukaemia (PML) protein in the sequential regulation of intracellular host immunity to HSV-1 infection. PLoS Pathog 14:e1006769. doi:10.1371/journal.ppat.1006769. 29309427 PMC5757968

[50] Sanjana NE, Shalem O, Zhang F 2014 Improved vectors and genome-wide libraries for CRISPR screening. Nat Methods 11:783–784. doi:10.1038/nmeth.3047. 25075903 PMC4486245

[51] Teo G, Liu G, Zhang J, Nesvizhskii AI, Gingras AC, Choi H 2014 SAINTexpress: improvements and additional features in Significance Analysis of INTeractome software. J Proteomics 100:37–43. doi:10.1016/j.jprot.2013.10.023. 24513533 PMC4102138

[52] Shannon P, Markiel A, Ozier O, Baliga NS, Wang JT, Ramage D, Amin N, Schwikowski B, Ideker T 2003 Cytoscape: a software environment for integrated models of biomolecular interaction networks. Genome Res 13:2498–2504. doi:10.1101/gr.1239303. 14597658 PMC403769

[53] MacLean B, Tomazela DM, Shulman N, Chambers M, Finney GL, Frewen B, Kern R, Tabb DL, Liebler DC, MacCoss MJ 2010 Skyline: an open source document editor for creating and analyzing targeted proteomics experiments. Bioinformatics 26:966–968. doi:10.1093/bioinformatics/btq054. 20147306 PMC2844992

[54] Vizcaino JA, Csordas A, del-Toro N, Dianes JA, Griss J, Lavidas I, Mayer G, Perez-Riverol Y, Reisinger F, Ternent T, Xu QW, Wang R, Hermjakob H 2016 2016 update of the PRIDE database and its related tools. Nucleic Acids Res 44:D447–D456. doi:10.1093/nar/gkv1145. 26527722 PMC4702828

[55] Liu H, Naismith JH 2008 An efficient one-step site-directed deletion, insertion, single and multiple-site plasmid mutagenesis protocol. BMC Biotechnol 8:91. doi:10.1186/1472-6750-8-91. 19055817 PMC2629768

[56] Katoh K, Rozewicki J, Yamada KD 2017 MAFFT online service: multiple sequence alignment, interactive sequence choice and visualization. Brief Bioinform bbx108. doi:10.1093/bib/bbx108. PMC6781576 28968734

[57] Yachdav G, Wilzbach S, Rauscher B, Sheridan R, Sillitoe I, Procter J, Lewis SE, Rost B, Goldberg T 2016 MSAViewer: interactive JavaScript visualization of multiple sequence alignments. Bioinformatics 32:3501–3503. doi:10.1093/bioinformatics/btw474. 27412096 PMC5181560

[58] Greco TM, Guise AJ, Cristea IM 2016 Determining the Composition and Stability of Protein Complexes Using an Integrated Label-Free and Stable Isotope Labeling Strategy. Methods Mol Biol 1410:39–63. doi:10.1007/978-1-4939-3524-6_3. 26867737 PMC4916643

[59] Wu G, Dawson E, Duong A, Haw R, Stein L 2014 ReactomeFIViz: a Cytoscape app for pathway and network-based data analysis. F1000Res 3:146. doi:10.12688/f1000research.4431.2. 25309732 PMC4184317

