# Supplementary figures and images for: Charge-Mediated Pyrin Oligomerization Nucleates Antiviral IFI16 Sensing of Herpesvirus DNA
Source: mBio. 2019 Jul 23;10(4):e01428-19. doi: 10.1128/mBio.01428-19 (PMC6650555; doi:10.1128/mBio.01428-19)

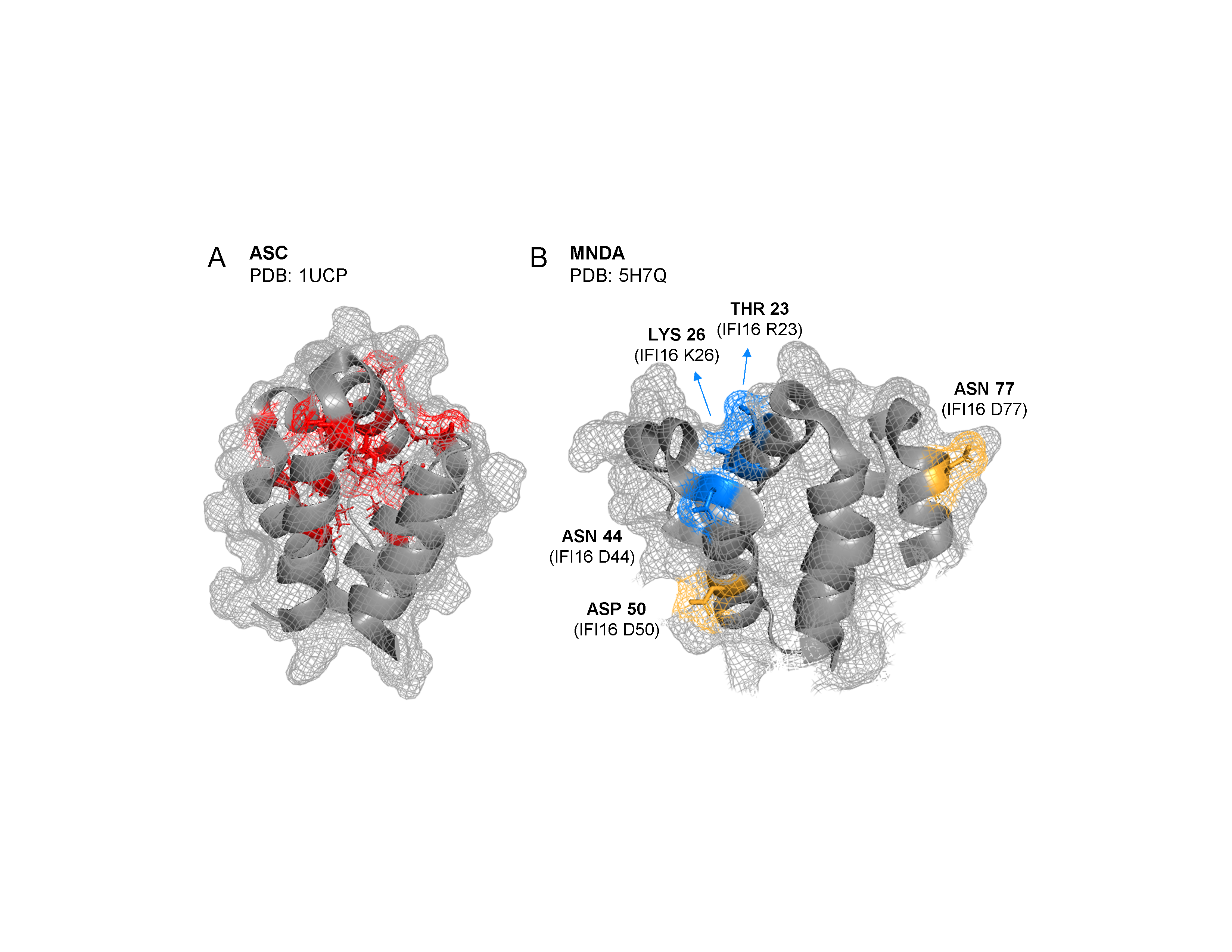

Supplement: FIG S1 [file mBio.01428-19-sf001.tif]

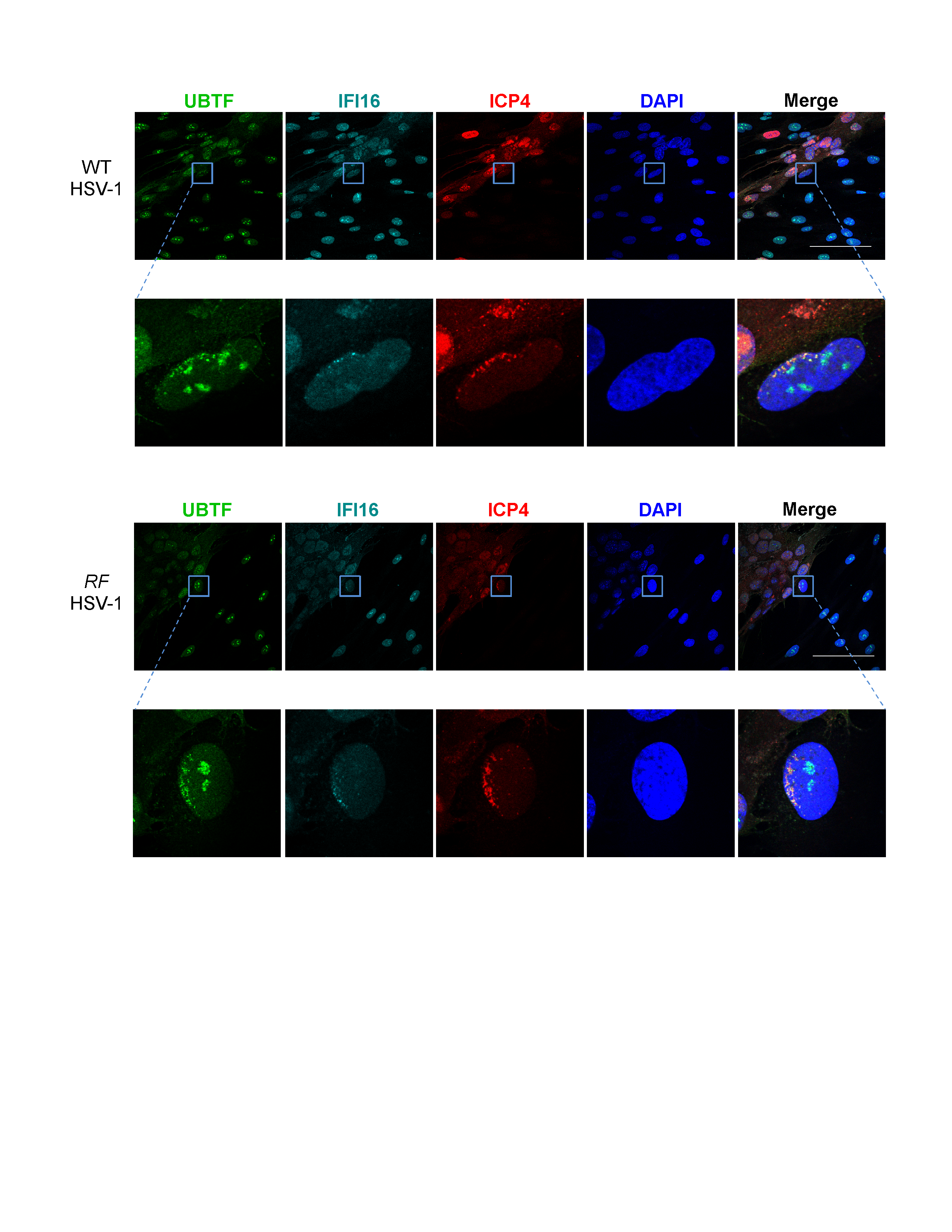

Supplement: FIG S3 [file mBio.01428-19-sf003.tif]
