# Supplementary material for: Charge-Mediated Pyrin Oligomerization Nucleates Antiviral IFI16 Sensing of Herpesvirus DNA
Source: mBio. 2019 Jul 23;10(4):e01428-19. doi: 10.1128/mBio.01428-19 (PMC6650555; doi:10.1128/mBio.01428-19)
Supplement: TEXT S1 [file mBio.01428-19-s0001.docx]

**Supplemental Materials and Methods**

**Antibodies**

The following antibodies were used for Western blotting and immunofluorescence microscopy: α-IFI16 (ab50004 and ab55328; Abcam), α-ICP0 (H1A027-100; Virusys Corporation), α-ICP27 (ab31631; Abcam), α-ICP8 (ab20194; Abcam), α-ICP4 (sc-69809; Santa Cruz Biotechnology), α-PAF1 (ab137519; Abcam), α-tubulin (T6199; Sigma-Aldrich), α-GFP (11814460001; Roche), α-PML (sc-9862; Santa Cruz Biotechnology), α-UBTF (H00007343-M04, Novus Biologicals; HPA006385, Atlas Antibodies), α-phospho-TBK-1 (D52C2; Cell Signaling Technology), α-TBK-1 (3504; Cell Signaling Technology), α-phospho-IRF3 (ab76493; Abcam), α-IRF3 (ab25950, Abcam). GFP-Trap_MA beads (Chromotek) were used for immunoaffinity purification of GFP and GFP-tagged IFI16.

**Virus Strains**

A bacterial artificial chromosome carrying the full HSV-1 (17+ strain) genome carried by *E. coli* strain GS1783 were a gift from Dr. Beatte Sodeik (Hannover Medical School, Hannover, Germany). Wild-type HSV-1 was produced by electroporation of pBAC-HSV-1 into Vero cells to harvest P0 stocks. The *ICP0-RF* HSV-1 mutant was a gift from Dr. Bernard Roizman (University of Chicago, Chicago, IL, USA) and Dr. Saul Silverstein (Columbia University, New York, NY, USA). The *d106* HSV-1 mutant was a gift from Dr. Neal DeLuca (University of Pittsburgh, Pittsburgh, PA).

**Plasmid construction**

All IFI16-GFP (PYD only and full-length) mutant constructs were generated by a site-directed mutagenesis method in the pEGFP-N1 plasmid for transient expression, pcDNA5/FRT/TO for inducible expression in HEK293 FlpIns, and LentiORF pLEX-MCS for stable expression in HFFs, as described (55). Chimeras of PYHIN protein constructs were generated via overlap-extension PCR and ligated into pEGFP-N1 using *XhoI* and *BamHI* restriction sites.

**Cell Line Construction, Transfections, and Lentivirus**

To generate cell lines, lentiviruses (pLEX-MCS and LentiCRISPRv2) were prepared according to protocols from the RNA interference (RNAi) Consortium. For CRISPR, candidate 20-bp guide RNA sequences were designed using the CRISPR Design Tool (<http://crispr.mit.edu/>) and delivered using the LentiCRISPRv2 vector (50) (Addgene plasmid 52961) from Dr. Feng Zhang. Guide RNA sequences are as follows: Scrambled (5’-GCTAAGATCTCGACAACACT-3’), IFI16 (5’-TGATGGAAGAAAAGTTCCG-3’), and UBTF (5’-CTGGAAATGGCCGCCCCCAA-3’). CRISPR-resistant IFI16 expression was achieved by expressing IFI16-GFP in pLEX-MCS with silent mutations in the IFI16 gene within the CRISPR guide RNA region: 5’-TCGGCGAGATCCTCCAGGGT-3’. Lentiviruses were generated in and harvested from HEK293T cells: packaging vectors psPAX2 and pMD2.G (VSV-G) were co-transfected with a lentiviral transfer vector in a ratio of 2.25:1.5:1.5 (psPAX2:pMD2.G:transfer vector) using XtremeGENE HP transfection reagent (Roche Diagnostics) at a ratio of 1:3 (µg DNA:µL XtremeGENE HP). Lentivirus was collected at 48, 72, and 96 hours post-transfection in 30% FBS DMEM and filtered through a 0.45 µm membrane. Supernatants containing lentivirus were laid onto a 5% sucrose cushion and subjected to ultracentrifugation (25,000 rpm, 2 hours, 4 °C with SW28 swinging bucket rotor [Beckman Coulter]). Lentiviral pellets were solubilized in PBS then flash-frozen in liquid nitrogen and stored at -80 °C until use.

**Cell Lines and Primary Cultures**

Primary human foreskin fibroblasts (HFFs), HFF-1, HEK293 FlpIn T-Rex, HEK293T, and U2OS cells were cultured using standard procedures in high glucose Dulbecco’s Modified Eagle’s Medium (Sigma-Aldrich) supplemented with 10% fetal bovine serum (Atlanta Biologicals), 1% penicillin, and 1% streptomycin.

To construct HFF-1 mutant cell lines, cells were transduced with lentivirus to express a CRISPR/Cas9/sgRNA cassette targeting IFI16 for two days. Positive transductants were selected with 2 µg/mL puromycin for five days before beginning another round of transduction with lentivirus carrying the CRISPR-resistant IFI16-GFP mutant constructs.

**Gene Sequence Alignment**

The PYD alignment was generated via MAFFT version 7 (<https://mafft.cbrc.jp/alignment/server/>) (56) and visualized using MSAViewer (57).

**SDS-PAGE and Western Blotting**

Cells were lysed in 1x Laemlli buffer (62.5 mM Tris-HCL, pH 6.8, 2% SDS (w/v), 10% glycerol (v/v), 0.02% bromophenol blue (w/v), 100 mM DTT) and boiled at 95 ^o^C for 5 minutes. For all PAGE, samples were electrophoresed on freshly prepared 10% acrylamide Tris/Glycine SDS-PAGE gels. Proteins were then electroblotted onto PVDF membrane before proceeding with standard Western blotting practices.

**RNA isolation and quantitative RT-PCR**

Total cellular RNA was purified with the RNeasy Mini kit (Qiagen) following the manufacturer’s instructions. Contaminating DNA was digested with DNAseI (Invitrogen) for 15 minutes at room temperature. RNA was reverse transcribed using the RETROscript Reverse Transcription kit (Life Technologies) and SuperScript IV First-Strand Synthesis Kit (ThermoFisher Scientific). Gene-specific primers and the SYBR green PCR master mix (Life Technologies) were used to quantify the resulting cDNA by qPCR on the AB7900HT and ViiA 7 real-time PCR systems (Applied Biosystems). Relative mRNA quantities were determined using the ∆∆CT method with β-actin as an internal control.

**Virus Infections and Progeny Virion Titers**

Both wild-type HSV-1 and the *ICP0-RF* virus stocks were generated in and titered using U2OS cells. To propagate virus, U2OS cell cultures were infected at low multiplicities of infection (MOI = 0.001) and incubated at 37 °C until 100% cytopathic effect (CPE) was observed (3-4 days). Both culture supernatant and cells were collected and buffered with MNT buffer (200 mM MES, 30mM Tris-HCl, 100 mM NaCl, pH 7.4). Supernatants were laid over 10% Ficoll cushions and subjected to ultracentrifugation (20,000 rpm, 2 hours, 4 °C with SW28 swinging bucket rotor [Beckman Coulter]) to concentrate virus. Cell-associated virus was collected by sonication and pooled with pelleted cell-free virus. Virus stock titers were determined by plaque assay on U2OS monolayers.

For all virus infections, viral stocks were diluted in 2% (v/v) FBS-containing DMEM to the indicated MOI and added to cell monolayers for 1 h at 37 ^o^C with intermittent rocking. After viral adsorption, cells were washed once with PBS, replenished with DMEM containing 10% (v/v) FBS, and incubated at 37 ^o^C for the indicated periods.

Target fibroblast cells were infected with either WT HSV-1 or RF HSV-1 at the indicated MOIs and time points. Cell-associated and cell-free virions were collected in pooled samples then titered on U2OS cell monolayers as described above.

**siRNA-Mediated Knockdowns**

Duplex sequences of siRNA were purchased from Sigma-Aldrich for the negative control (MISSION siRNA Universal Negative Control #1; Sigma-Aldrich #SIC001), and from Dharmacon for PAF1-1 (SMARTpool: ON-TARGETplus L-020349-01-0005), Sigma Aldrich for PAF1-2 (SASI_Hs01_00045420) and for PAF1-3 (FAST siRNA, 5’ – AAGAGACGUUGAAGAAACGAA – 3’). Transfections in HFFs were performed with Lipofectamine RNAiMAX transfection reagent (ThermoFisher Scientific) following the manufacturer’s protocol. After transfection, cells were recovered for 24 h in 10% FBS DMEM and immediately either infected with the indicated HSV-1 conditions or processed for Western blot analysis. Upon infection at 0 hpi, an additional round of siRNA transfection was conducted.

**Fluorescence Imaging**

For immunofluorescence imaging, fixed and permeabilized cells were sequentially probed with primary antibody and Alexa Fluorophore-conjugated secondary antibody (Life Technologies), diluted in blocking buffer, for 1 hour. Nuclei were stained using 1 µg/ml 4,6-diamidino-2-phenylindole in PBS for 10 min. All steps were carried out at room temperature. Microscopy was performed using a Leica TC SP5 confocal microscope (Leica Microsystems) and an inverted fluorescence confocal microscope (Nikon Ti-E) equipped with a Yokogawa spinning disc (CSU-21) and digital camera (Hamamatsu ORCA-Flash TuCam).

For live-cell imaging of UBTF and PAF1 microscopy experiments in HFFs, cells were seeded on uncoated 35mm glass bottom dishes (MatTek). For all other cells, fixed-cell imaging was performed. Cells were seeded on glass coverslips placed in 6-well dishes (Biolite). In all cases, cells were given at least 24 hours to attach prior to experimental manipulation. For live-cell imaging experiments, cells were maintained at 37^o^C and 5% CO_2_ using an environmental control chamber. For all experiments, cells were imaged with either 60x or 100x oil immersion objectives.

**Cross-linking assay to assess IFI16 oligomerization**

HEK293T cells were transfected with the indicated IFI16-GFP constructs for 12 h, scraped, and lysed on ice for 30 min in lysis buffer (20 mM K-HEPES pH 7.4, 0.1 M potassium acetate, 2 mM MgCl_2_, 0.1% (v/v) Tween-20, 1 μM ZnCl_2_, 1 μM CaCl_2_, 1% Triton X-100, 200 mM NaCl, 2.5 Unit/mL Benzonase (Pierce) and 1x Halt Protease and Phosphatase Inhibitor cocktail. Upon clarification by centrifugation at 3,000 ×g, freshly prepared glutaraldehyde was added to a final concentration of 5 mM and incubated with lysates for 7 min at room temperature. The cross-linking reaction was quenched by the addition of 50 mM glycine and SDS sample buffer. A parallel set of transfected cells were kept un-cross-linked and lysed in SDS sample buffer. Samples were boiled and analyzed via SDS-PAGE and western blot using GFP antibody.

**Immunoaffinity Purifications of IFI16-GFP and identification of interactions by mass spectrometry**

Immunoaffinity purifications of IFI16-GFP protein complexes were carried out using 20 µL of GFP-Trap_MA GFP antibody-coupled magnetic beads (Chromotek) per sample. Cells were washed once with cold PBS then scraped for collection. After pelleting by centrifugation (300 x *g*), cells were resuspended in a small volume of freezing buffer (20mM Na-HEPES, 1.2% polyvinylpyrrolidone, 1x PIC/PhIC [Thermo Scientific], pH 7.4) and stored overnight at -80 °C. To lyse cells, lysis buffer (20 mM K-HEPES, pH 7.4, 0.11 M KOAc, 0.1% Tween-20 (v/v), 200 mM NaCl, 0.6% Triton X-100, 1x PIC/PhIC, 100 U/mL Benzonase [Pierce]) was added and cells were kept on ice for 30 minutes with vortexing every 10 minutes. Cell lysates were clarified by centrifugation at 8,000x*g* for 10 minutes at 4 °C.

IPs were performed for 1 hour at 4 °C then beads were washed three times with wash buffer (20 mM K-HEPES, pH 7.4, 0.11 M KOAc, 0.1% Tween-20 (v/v), 200 mM NaCl, 0.6% Triton X-100). Proteins were eluted in 1x TES (1% sodium dodecyl sulfate, 5 mM EDTA, 10 mM Tris-HCl 7.4) by incubating at 70 °C for 10 min then vigorously vortexing for 30 seconds. Samples were then frozen at -20 °C or immediately prepared for Western blotting or mass spectrometry analysis.

**Sample Preparation for Mass Spectrometry**

IP eluate samples were first reduced and alkylated in 5 mM TCEP, 15mM chloroacetamide for 20 minutes at 70 °C. For HEK293T IFI16-GFP IPs, protein digestion with 0.25 µg MS-grade trypsin (Pierce) was performed via overnight filter aided sample preparation (FASP) with Amicon Ultra-0.5 filters as previously described (58), followed by desalting with StageTips with SDB-RPS membranes. For IFI16-GFP IPs in HFF-1 mutants, digestion was performed with 0.75 µg trypsin and suspension trapping columns (S-Trap, Protifi) for 1 hour, according to the manufacturer’s instructions. Following either digestion, peptide eluates were resuspended in 10 µL of 1% FA, 1% ACN.

**Mass Spectrometry Acquisition**

Peptides were analyzed by nano-liquid chromatography coupled to tandem mass spectrometry with a Q Exactive HF Hybrid Quadrupole-Orbitrap instrument (Thermo Scientific) using data-dependent acquisition (DDA) or parallel-reaction monitoring (PRM) modes. Peptides (2 µL injections) were separated with a 3% solvent B to 30% solvent B gradient (solvent A: 0.1% FA, solvent B: 0.1% FA, 97% ACN) over 60 min at a flow rate of 250 nL/min on an EASYSpray C18 column (75 µm x 50 cm) heated to 50 °C. For DDA, the full scan range was set to 350-1800 m/*z* at 120,000 resolution and recorded in profile. The top 15 most intense precursors were subjected to HCD fragmentation – normalized collision energy (NCE) of 28 – for MS^2^ analysis at 30,000 resolution with automatic gain control (AGC) target set to 1E5, 120 ms maximum injection time (MIT), and an isolation window of 1.2 m/*z*. For PRM, the instrument was set to 15,000 resolution, AGC target was set to 1E5, MIT of 100 ms, 0.8 m/*z* isolation window, and NCE set to 27. An MS^1^ scan was acquired after every 20 PRM scans. MS^1^ acquisition was performed at 15,000 resolution, 25 ms MIT, and full scan range of 350-1800 m/*z*.

**Analysis of MS Data and Assembly of IFI16 Interaction Network**

Tandem MS spectra collected from DDA mode were analyzed by Proteome Discoverer v2.2 (Thermo Fisher Scientific). MS spectra were searched using the Sequest HT algorithm against a UniProt human database containing herpesvirus sequences and common contaminants (22,349 sequences, downloaded 2016-04). The Spectrum Files RC node was used to perform offline mass recalibration and the Minora Feature Detector node was used for label-free MS^1^ quantitation. The search required fully tryptic peptides with a maximum of two missed cleavages, 5 ppm precursor mass tolerance, and a 0.02 Da fragment ion mass accuracy. Posttranslational modifications (PTMs) including static carbamidomethylation of cysteine, dynamic oxidation of methionine, dynamic deamidation of asparagine, dynamic loss of methionine plus acetylation of the protein N-terminus, and dynamic phosphorylation of serine, threonine, and tyrosine were all allowed. The Percolator node was then used to perform peptide spectrum match (PSM) validation and the ptmRS node was used for assigning PTM sites. PSMs were assembled into peptide and protein identifications with a false discovery rate of less than 1% for both the peptide and protein level with at least 2 unique peptides identified per protein. The MS^1^ protein abundances were normalized to the median protein abundance value for the bait IFI16 before filtering for specific interactions.

Identified proteins were filtered for specificity with the Significance Analysis of INTeractome express (SAINTexpress) algorithm (33, 51) using MS^1^ intensity values, and a cutoff score of 0.80 was used for determining specificity (Table S1). Then, proteins were cross-referenced against the CRAPome repository (34) to further filter for specific interactions, and proteins appearing in more than 20% of negative-control experiments were removed (Table S2). Lastly, proteins were filtered by known subcellular localization (UniProt) to only include those residing in the nucleus. The specificity-filtered proteins were then used to generate an interaction network using the ReactomeFIPlugIn (v. 7.1.0) (59) within the Cytoscape software (v. 3.7.0) (51), and proteins were categorized by Gene Ontology. Nodes were colored by enrichment with R23K IFI16-GFP (charge mimic) vs R23Q IFI16-GFP (structural mimic) and organized in the network by gene ontology categories.

**PRM Library and Quantitative Analysis of Data**

PRM assays were designed and analyzed using the Skyline Daily software (53). Proteotypic peptides for each protein of interest were selected and the elution and fragmentation profiles were experimentally determined for each targeted peptide (Table S3). Peptide abundance was quantified using the summed area under the curve of 3-5 fragment ions per peptide. Peptide abundance values were scaled to the wild type condition for each peptide then normalized by the average IFI16 bait abundance in each IP, as quantified by three peptides for IFI16. One-tailed Student’s t-tests were performed using the software Prism v5.04.
